# Berberine governs NOTCH3/AKT signaling to enrich lung-resident memory T cells during tuberculosis

**DOI:** 10.1371/journal.ppat.1011165

**Published:** 2023-03-07

**Authors:** Isha Pahuja, Kriti Negi, Anjna Kumari, Meetu Agarwal, Suparba Mukhopadhyay, Babu Mathew, Shivam Chaturvedi, Jaswinder Singh Maras, Ashima Bhaskar, Ved Prakash Dwivedi

**Affiliations:** 1 Immunobiology Group, International Centre for Genetic Engineering and Biotechnology, Aruna Asaf Ali Marg, New Delhi, India; 2 Department of Molecular Medicine, Jamia Hamdard University, New Delhi, India; 3 Department of Molecular and Cellular Medicine, Institute of Liver and Biliary Sciences, New Delhi, India; Portland VA Medical Center, Oregon Health and Science University, UNITED STATES

## Abstract

Stimulation of naïve T cells during primary infection or vaccination drives the differentiation and expansion of effector and memory T cells that mediate immediate and long-term protection. Despite self-reliant rescue from infection, BCG vaccination, and treatment, long-term memory is rarely established against *Mycobacterium tuberculosis* (*M*.*tb*) resulting in recurrent tuberculosis (TB). Here, we show that berberine (BBR) enhances innate defense mechanisms against *M*.*tb* and stimulates the differentiation of Th1/Th17 specific effector memory (T_EM_), central memory (T_CM_), and tissue-resident memory (T_RM_) responses leading to enhanced host protection against drug-sensitive and drug-resistant TB. Through whole proteome analysis of human PBMCs derived from PPD^+^ healthy individuals, we identify BBR modulated NOTCH3/PTEN/AKT/FOXO1 pathway as the central mechanism of elevated T_EM_ and T_RM_ responses in the human CD4^+^ T cells. Moreover, BBR-induced glycolysis resulted in enhanced effector functions leading to superior Th1/Th17 responses in human and murine T cells. This regulation of T cell memory by BBR remarkably enhanced the BCG-induced anti-tubercular immunity and lowered the rate of TB recurrence due to relapse and re-infection. These results thus suggest tuning immunological memory as a feasible approach to augment host resistance against TB and unveil BBR as a potential adjunct immunotherapeutic and immunoprophylactic against TB.

## Author summary

In response to primary infections or vaccination, the host immune system elicits robust effector and memory responses to facilitate immediate pathogen clearance and to establish long-term protection. In this study, we have ascertained the prospects of potent immunomodulator berberine (BBR) in instigating host protective immunological memory responses during tuberculosis (TB). BBR-induced glycolytic flux stimulates pro-inflammatory immune responses against Mycobacterium tuberculosis (*M*.*tb*). BBR further prompted NOTCH-mediated AKT inhibition and activation of FOXO1, STAT3, STAT4, BLIMP-1, and NFκB signaling thereby enriching *M*.*tb*-specific resident memory T cell population in the distinct murine TB disease models and human CD4+ T cells. These results project BBR as an adjunct immunotherapeutic and immunoprophylactic against TB.

## Introduction

In spite of being avertable and treatable, tuberculosis (TB) caused by *Mycobacterium tuberculosis* (*M*.*tb*) is the major cause of mortality and morbidity among infectious diseases. Globally 10 million people were diseased with TB and an aggregate of 1.3 million people passed away in 2020 itself [1]. Furthermore, almost one-fourth of humankind is infected asymptomatically (latently) with *M*.*tb*, with a 5–15% risk of progressing into clinical manifestations [1].

Existing anti-tubercular treatment (ATT) comprising of assorted anti-mycobacterial drugs can only exterminate active, drug-sensitive strains of *M*.*tb*. However, failure to complete the extensive TB restraint approach, directly observed treatment short-course (DOTS) frequently brings about the emergence of multi-drug resistant (MDR) and extensively-drug resistant (XDR) strains. Moreover, DOTS therapy instigates severe toxicity and impairment of host immune responses. For instance, isoniazid (INH) usage leads to the cessation of antigen-responding CD4^+^ T lymphocytes, which results in a heightened risk of reactivation and reinfection with *M*.*tb* [2]. Further inefficacy of the only available vaccine *M*. *bovis* bacille Calmette-Guérin (BCG) to prevent adult pulmonary TB makes it a requisite to employ appropriate strategies to augment the host control of *M*.*tb* infection [3].

Subsequent to infection, *M*.*tb* is phagocytosed by antigen-presenting cells (APCs) that participate in the extermination of internalized pathogens, promote activation of T lymphocytes and stimulate protective pro-inflammatory cytokines such as IFNγ and IL17 [4–6]. The immunological response in TB is extremely complex and the fate of infection is governed predominantly by subsets of T lymphocytes [7]. For instance, stimulation of T helper 2 (Th2) cells and regulatory T cells (Tregs) result in the advancement of disease by hampering protective Th1 responses [8,9,10] while Th1/Th17subsets are associated with host protective immune responses [11]. Nonetheless, these cytokine responses decline post *M*.*tb* clearance and hence, subsets of memory T cells play a crucial role in providing long-term protection in TB [11]. T cell receptor (TCR) signaling following antigen stimulation along with cytokine environment regulates and shapes host memory responses [12]. Sustained AKT activation following TCR stimulation drives terminal T cell differentiation [13] probably by targeting FOXO proteins [14,15]. JAK/STAT pathways also influence the differentiation of naïve T cells into memory subsets [16]. Further, STAT4 and Blimp1 transcription factors are known to regulate resident memory responses at the local site of infection. T effector memory (T_EM_) cells provoke Th1 type cytokines and protect against acute *M*.*tb* infections whereas, T central memory (T_CM_) can give rise to T_EM_ during disease progression, direct cell-mediated immunity for bacterial clearance and sustain long-term memory responses [17,18]. Hence, a strengthened T_CM_ and T_EM_ population is vital for the continuation of long-term protective immune responses [19]. Apart from these, tissue-resident memory T cells (T_RM_) localized at distinctive sites of infections like lung and spleen are linked with positive medical consequences and host protective responses [20].

Mostly host immune responses elicited in response to *M*.*tb* can successfully contain pathogen [4] but complete sterility is not attained. Hence, a novel immunomodulatory approach is necessitated to boost current therapeutics to ease up drug regimen, lessen therapy-induced adversities and intensify anti-mycobacterial effects [21,22,23]. BBR (C_20_H_18_NO_4_^+^) a bioactive isoquinoline alkaloid is known for diverse therapeutic effects. Administering BBR adjunct to therapeutics can induce hepatoprotective effects by modulating inflammatory responses [24]. BBR can induce protection against INH-associated inflammation, oxidative stress and liver damage in rats [25]. BBR has shown affirmative outcomes *in vitro* against clinical drug-resistant bacterial strains [26], and *in vivo* against drug-sensitive *M*.*tb* with limited insight into the mechanism of protection[27]. In this study, we have investigated the anti-mycobacterial potential of BBR against pathogenic laboratory strain H37Rv and drug-resistant clinical isolates of *M*.*tb ex vivo* and in the murine model of TB. We observed that BBR significantly lowered the bacterial burden in the lungs and the spleen of *M*.*tb* infected mice in solitary or in combination with the first-line anti-TB drug INH primarily by boosting the protective host immune responses such as macrophage activation, Th1/Th17 polarization, memory T cell enhancement and pro-inflammatory cytokine responses. NOTCH mediated AKT inhibition and activation of FOXO1, STAT3, STAT4, BLIMP-1 and NFκB signaling following BBR treatment led to a profound induction of adaptive memory in human CD4^+^ T cells and in mice model. These immunomodulatory properties of BBR were also exploited to increase the vaccine efficacy of BCG. Furthermore, induction of superior memory responses significantly lowered the recurrence of TB due to re-activation and re-infection. Overall, these results propound BBR as an attractive adjunct immunotherapeutic and immunoprophylactic against TB.

## Results

### BBR enhances host resistance against drug-susceptible and drug-resistant strains of *M*.*tb*

**Fig 1:**
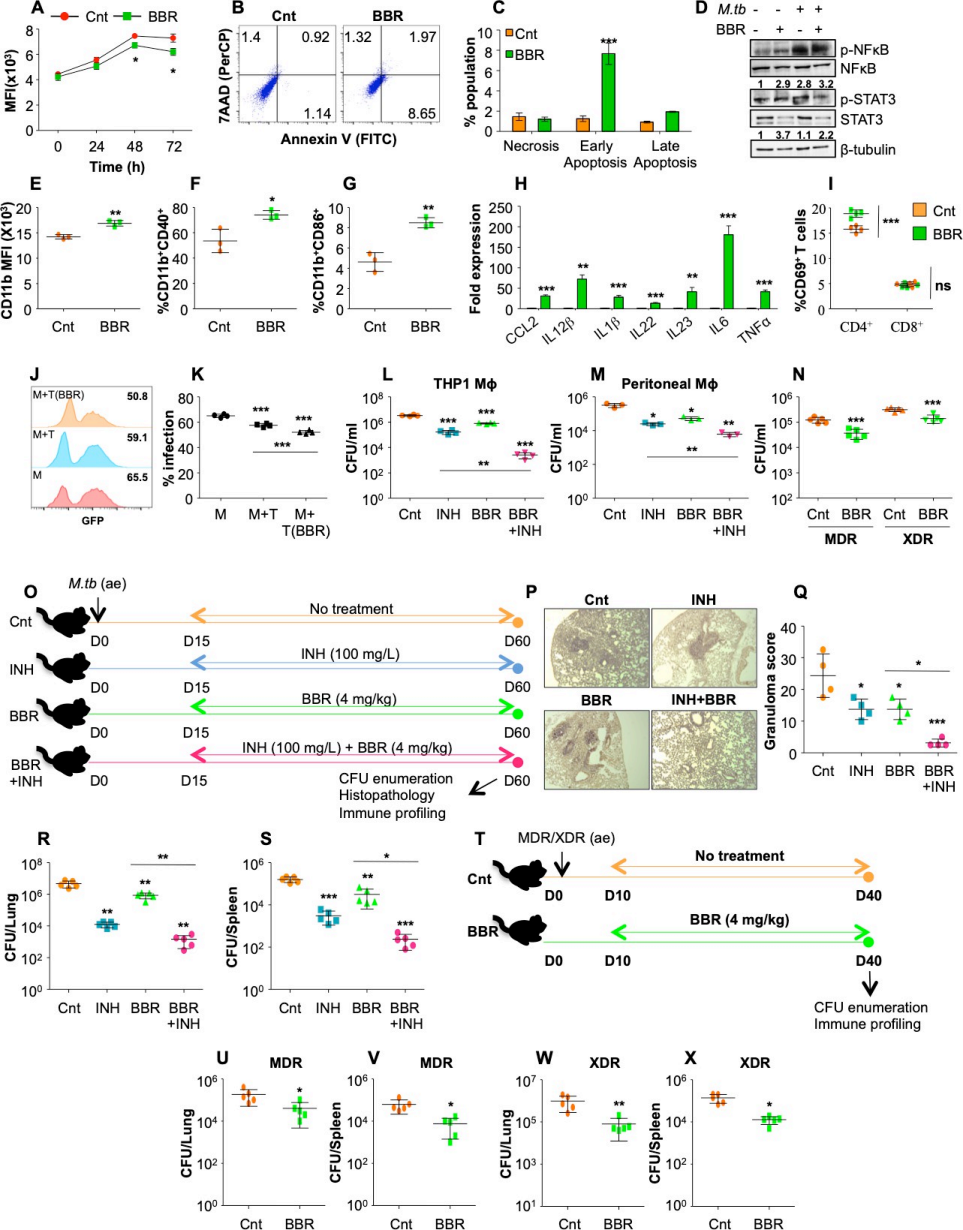
BBR treatment enhances host resistance against drug-sensitive and drug–resistant TB. **(A)** Mouse peritoneal macrophages were infected with GFP expressing H37Rv (Rv-GFP) at 1:10 MOI followed by treatment with BBR (20 μg/ml). At different time points, cells were analysed by flow cytometry. Graph represents the GFP fluorescence at indicated time points with and without BBR treatment. **(B-C)** Mouse peritoneal macrophages were infected with *M*.*tb* at MOI of 1:10 followed by treatment with BBR (20 μg/ml) for 48 h followed by apoptosis analysis via flow cytometry. **(B)** Representative dot plots and **(C)** Percentage of apoptotic cells with and without BBR (20μg/ml) treatment. **(D)** Immunoblots depicting the phosphorylation of indicated transcription factors (NFkB and STAT3) in uninfected and infected mouse peritoneal macrophages with or without BBR treatment. **(E-G)** Infected murine peritoneal macrophages were surface stained with antibodies against CD11b (APC/Cy7), CD40 (PE) and CD86 (PerCPCy5.5) followed by flow cytometry. **(E)** Expression of CD11b on the surface of infected macrophages. Percentage of **(F)** CD11b^+^CD40^+^ and **(G)** CD11b^+^CD86^+^ infected macrophages with and without BBR treatment. **(H)** Expression of chemokines and cytokines in *M*.*tb* infected macrophages at 24h pi with and without BBR (20 μg/ml) treatment. **(I)** Percentage of CD4^+^ and CD8^+^ T cells expressing CD69 in the infected and BBR (10μg/ml) treated splenocytes. **(J)** Representative overlay plots and **(K)** percentage of RvGFP infected macrophages co-cultured with *M*.*tb* specific and BBR treated splenocytes. **(L)** PMA- activated THP1 macrophages were infected with H37Rv at 1:10 MOI followed by treatment with INH (1 μg/ml), BBR (20 μg/ml) or both for 48 h pi after which cells were lysed for CFU enumeration. **(M)** Experiment L was repeated in mouse peritoneal macrophages. **(N)** Mouse peritoneal macrophages were infected with MDR (Jal 2261) and XDR (MYC431) clinical strains of *M*.*tb* followed by treatment with 20 μg/ml of BBR. Cell lysates were plated for CFU enumeration 48 h pi. **(O)** Schematic representation of the murine model of infection. C57BL/6 mice were infected with low dose of H37Rv (~110 CFU per lung) and after 15 days of disease establishment, mice were treated with either INH (100 mg/L), BBR (4 mg/kg) or both for 45 days followed by CFU enumeration and immune profiling. **(P)** Histopathology of infected lungs with arrows indicating the granulomatous lesions. **(Q)** Immunopathology score of the infected lungs. Bacterial burden in the **(R)** lungs and the **(S)** spleen of infected animals. **(T)** Diagrammatic representation of the infection model. Bacterial burden in **(U)** the lungs and **(V)** the spleen of mice infected with MDR strain of *M*.*tb*. Bacterial load in **(W)** the lungs and **(X)** the spleen of mice infected with XDR strain of *M*.*tb*. Data is representative of two independent experiments. The data values represent mean ± SD (n = 3–5). *p<0.05, **p<0.005, ***p<0.0005.

Numerous studies have evaluated the therapeutic potential of BBR against diverse ailments. However, the effectiveness of BBR against *M*.*tb* infection is yet to be uncovered. Since BBR has been shown to possess weak anti-bacterial activity [28], we foremost aimed to determine its anti-mycobacterial activity against different drug-sensitive and -resistant strains of *M*.*tb*. Consistent with the previous studies, BBR displayed bacterial toxicity at ≤ 50 μg/ml against all the strains tested **(S1A–S1C Fig)** however 20 μg/ml of BBR treatment which displayed no toxicity in the mouse peritoneal macrophages **(S1D and S1E Fig)** significantly decreased the intracellular *M*.*tb* growth **(Fig 1A)** indicating the immunomodulatory effects of BBR on host macrophages. With no variable effect on ROS generation **(S1F and S1G Fig)**, BBR treatment significantly induced apoptosis in uninfected (**S1H Fig**) and *M*.*tb* infected macrophages **(Fig 1B and 1C)** and led to significant activation of transcription factors (NFkB and STAT3) which play a crucial role in combating TB **(Fig 1D).** Further, BBR treatment significantly enhanced the expression of CD11b and co-stimulatory molecules CD40 and CD86 on the surface of infected macrophages **(Fig 1E–1G)**. Moreover, BBR treated macrophages also displayed increased expression of M1 specific pro-inflammatory cytokines **(Fig 1H)**. Furthermore, immunomodulatory effect of BBR was consistent in T cells isolated from infected mice wherein the percentage of CD69^+^ activated CD4^+^ T lymphocytes was significantly enriched upon BBR treatment **(Fig 1I)**. Co-culturing infected macrophages with BBR primed T cells demonstrated significantly reduced intracellular bacterial burden **(Fig 1J and 1K)** as compared to infected macrophages as well as untreated T cells co-cultured with macrophages. Few piecemeal studies have reported BBR as an efflux pump inhibitor and thereby is known to increase the intracellular concentration of the antibiotics [29]. We investigated whether BBR co-treatment increased the killing potential of INH. With no significant effect *in vitro* **(S1G Fig)** BBR co-treatment significantly reduced the bacterial burden in the INH treated macrophages as compared to INH treatment alone in both human **(Fig 1L)** and mice derived macrophages **(Fig 1M)**. BBR also reduced the intracellular load of MDR and XDR strains of *M*.*tb* **(Fig 1N)** emphasizing that the immunomodulatory potential of BBR is not restricted to drug-sensitive strain of *M*.*tb*. To further corroborate the outcomes of *ex vivo* experiments, C57BL/6 mice were infected with a low dose (∼110 CFU) of *M*.*tb* H37Rv and left untreated or treated with BBR (4mg/kg) either alone or in combination with INH (100mg/L) for 45 days followed by CFU enumeration and immune profiling **(Fig 1O)**. As compared to control or INH treated infected lungs, BBR treatment significantly reduced the extent of granulomatous inflammation alone and in combination with INH **(Fig 1P and 1Q)**. Further, BBR treatment significantly reduced the bacterial burden in the lungs and the spleen of infected mice as compared to the control group **(Fig 1R and 1S)**. Interestingly, BBR co-treatment significantly enhanced the anti-tubercular potential of INH **(Fig 1R and 1S)**. These results demonstrate the adjunct potential of BBR along with INH against *M*.*tb* H37Rv. Since drug-resistant variants are one of the major contributors of global TB pandemic, we ascertained the efficacy of BBR against MDR and XDR TB **(Fig 1T)**. Interestingly, BBR treatment significantly lowered the bacterial burden of both the drug-resistant strains tested in the lungs and spleen of mice **(Fig 1U–1X)**.

### BBR strengthens the host protective immune responses against TB

**Fig 2:**
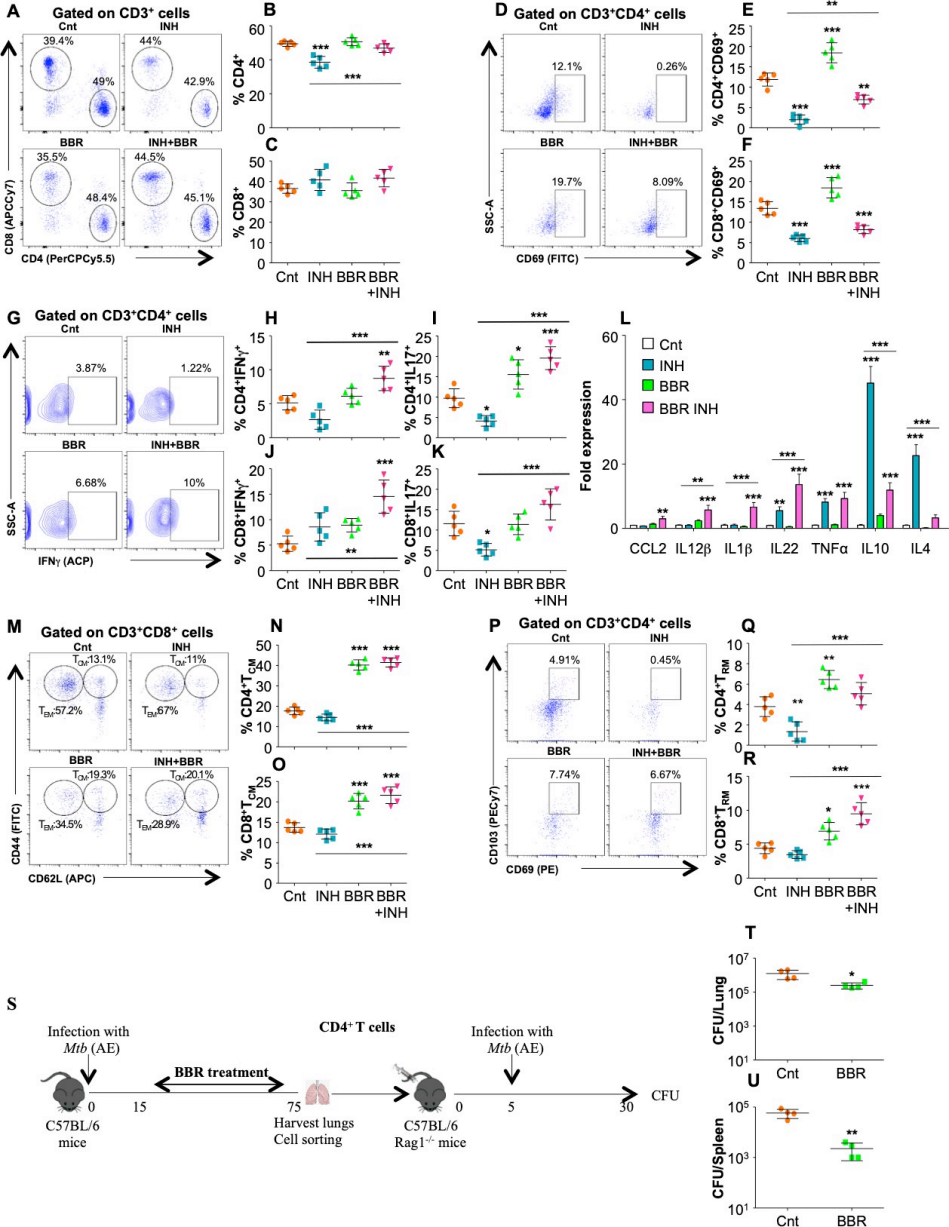
BBR strengthens *M*.*tb*-specific T cell responses during TB treatment. Single cell suspensions generated from the infected lungs were *ex vivo* stimulated with *M*.*tb* complete soluble antigen (CSA) for 16 h followed by surface staining with antibodies against CD3 (Pacific Blue), CD4 (PerCPCy5.5), CD8 (APCCy7) and CD69 (FITC) followed by flow cytometry. **(A)** FACS scatter dot plots and percentage of **(B)** CD4^+^ and **(C)** CD8^+^ T cells in the infected lungs. **(D)** Representative FACS dot plot and the percentage of **(E)** CD4^+^CD69^+^ and **(F)** CD8^+^CD69^+^ T cells in the infected lungs. **(G-K)** After stimulation with CSA, the lung cells were treated with monensin and brefeldin A for 2 h and surface stained with α-CD3 (Pacific Blue), α-CD4 (PerCPCy5.5) and α-CD8 (APCCy7) followed by intracellular staining with α-IFNγ (APC) and α-IL17 (PECy7) and flow cytometry. **(G)** Representative dot plots and percentage of **(H)** CD4^+^IFNγ^+^, **(I)** CD4^+^IL17^+^, **(J)** CD8^+^INFγ^+^ and **(K)** CD8^+^IL17^+^ cells in the infected lungs. **(L)** Fold expression of cytokines in the lungs of infected, INH and BBR treated splenocytes. **(M-R)** To determine the frequency of central memory and resident memory T lymphocytes, *ex vivo* stimulated lung cells were surface stained with α-CD3 (Pacific Blue), α-CD4 (PerCPCy5.5), α-CD8 (APCCy7), α-CD69 (PE), α-CD103 (PECy7), α-CD62L (APC) and α-CD44 (FITC) followed by flow cytometry. **(M)** Representative FACS dot plots and **(N)** the percentage of CD4^+^T_CM_ (CD62L^HI^CD44^HI^) cells, T cell subset. **(O)** Percentage of CD8^+^TCM (CD62L^HI^CD44^HI^) cells, in the infected lungs with or without drug treatment. **(P)** Representative scatter dot-plot images and percentage of **(Q)** CD4^+^TRM and **(R)** CD8^+^TRM cells in the lungs of infected mice. **(S)** Schematic representation of adoptive transfer experiment. CFU enumeration after 21 days of adoptive transfer in **(T)** the lungs and **(U)** the spleen of Rag-/- mice. Data is representative of two independent experiments. The data values represent mean ± SD (n = 5). *p<0.05, **p<0.005, ***p<0.0005.

To comprehend the immunomodulatory properties of BBR, we profiled the innate and adaptive immune cell populations driving host protection in both the lungs and the spleen of infected mice. Increased percentage of CD11b^+^ and CD11c^+^ cells with enhanced expression co-stimulatory molecules CD80 and CD86 was observed in the lungs **(S2A–S2H Fig)** and the spleen **(S2I–S2P Fig)** of infected mice treated with BBR indicating significant innate cell stimulation which plays a crucial role in the activation of adaptive immune responses. Although BBR treatment did not change the percentage of CD4^+^ and CD8^+^ T cells **(Fig 2A–2C)**, in combination with INH, BBR convalesced INH induced reduction in percentage of CD4^+^ T cells **(Fig 2B)** in the lungs of infected mice. BBR treatment significantly induced the activation of T cell subsets as the expression of early activation marker CD69 on these cells was significantly heightened in the lungs **(Fig 2D–2F)** and the spleen **(S3A–S3E Fig)** of infected mice.

Hence, it can be inferred that BBR treatment extensively strengthens antigen processing and presentation by APCs and consistently promotes activation of T lymphocytes to impart protection against *M*.*tb* infection. Furthermore, BBR treatment advanced differentiation of CD4^+^ and CD8^+^ into protective Th1 and Th17 subsets in the lungs of infected mice. This was evident by significant increase in IFNγ and IL17 producing T cell subsets in both the lungs **(Fig 2G–2K)** and the spleen **(S3F–S3J Fig)** of BBR and INH treated mice. Furthermore, BBR treatment considerably enriched host-protective chemokines and cytokines such as TNFα, IL1β, IL22 etc., and subsided the effect of INH induced anti-inflammatory cytokines such as IL10 and IL4 in co-treated mice **(Fig 2L).** We further investigated the impact of BBR treatment on the induction of prolonged immune protection, which is mediated by memory subsets of adaptive immunity. The lungs of BBR treated mice revealed high frequency of T_CM_ cells **(Fig 2M–2O)**. Similar trend was observed in the spleen of BBR treated mice **(S4A–S4C Fig)**. Furthermore, the percentage of resident memory T cells (T_RM_) which evolve from disseminating effector memory T cells (T_EM_), remain confined to the tissues and play a key role in stimulating adaptive immune response at the tissue specific sites was considerably enhanced in the lungs **(Fig 2P–2R)** and in the spleen of infected mice **(S4D–S4F Fig)**. Furthermore, to strengthen our findings regarding positive immunomodulatory effects of BBR treatment on CD4^+^ T cells, we performed adoptive transfer experiment in Rag^-/-^ mice **(Fig 2S)**. Adoptive transfer of CD4^+^ T cells from BBR treated infected mice into Rag^-/-^ mice significantly reduced bacterial burden in the lungs **(Fig 2T)** and the spleen **(Fig 2U)** of Rag^-/-^ mice upon *M*.*tb* infection concluding that BBR exerts host-protective effects by enriching *M*.*tb* specific CD4^+^ T cell responses.

### BBR enriches pathways associated with establishment of T_RM_ in human PBMCs

**Fig 3:**
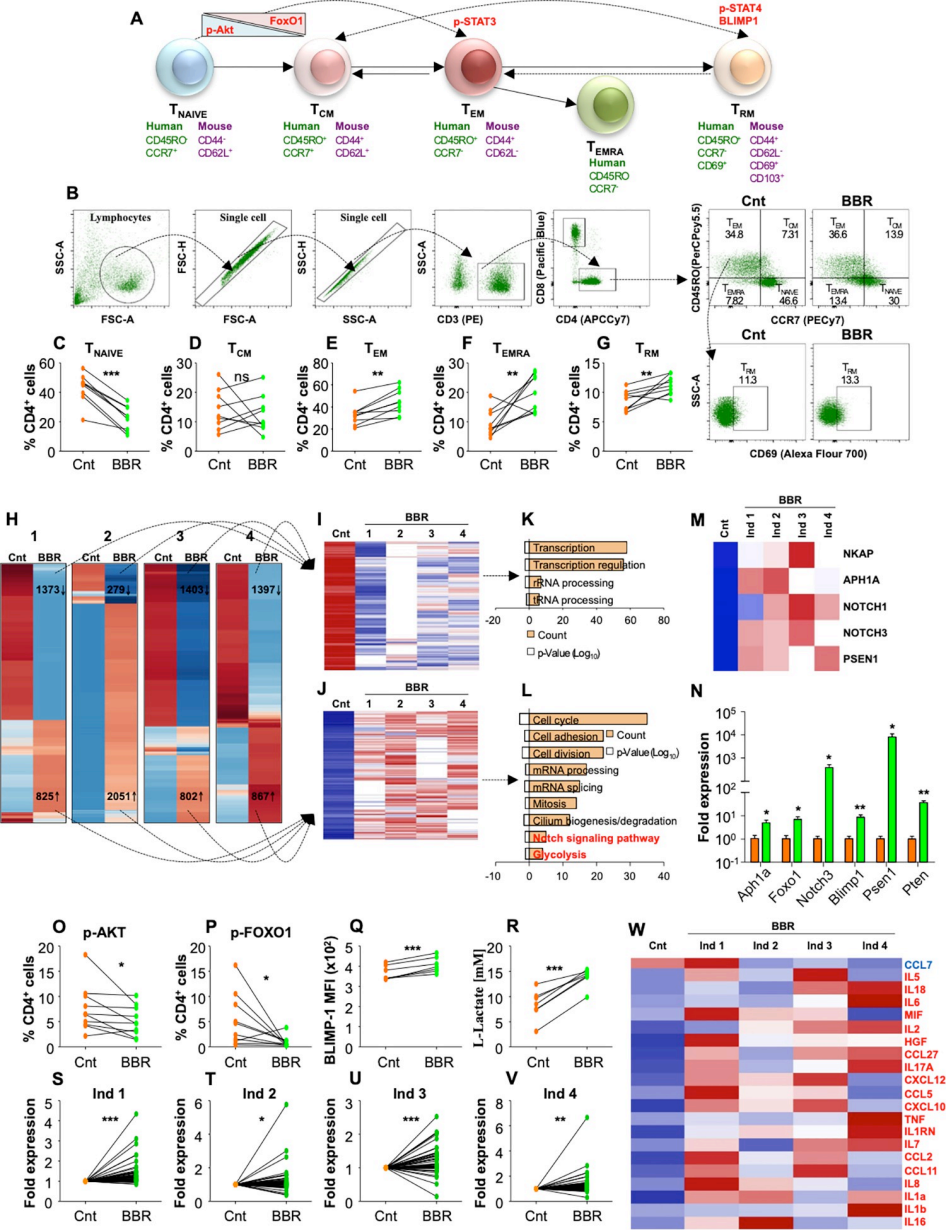
BBR treatment enriches human CD4^+^ memory T cells by regulating NOTCH/PTEN/Akt/FOXO1 pathway. **(A)** Schematic representation of T cell differentiation into T_EM_, T_CM_ and T_RM_ memory subsets. **(B-G)** Human PBMCs isolated from 7 PPD^+^ healthy individuals were ex vivo stimulated with CSA and treated with BBR (10 μg/ml) for 48 h followed by surface staining with α-CD3 (PE), α-CD4 (APCCy7), α-CD8 (Pacific Blue), α-CD45RO (PerCPCy5.5), α-CCR7 (PECy7) and α-CD69 (Alexa Flour 700) **(B)** Gating strategy employed to depict the different memory T cell subsets. Percentage of **(C)** CD4^+^ T _NAIVE_ cells, **(D)** CD4^+^ T_CM_ cells, **(E)** CD4^+^ T_EM_ cells, **(F)** CD4^+^ T_EMRA_ cells and **(G)** CD4^+^ T_RM_ cells. **(H)** Whole proteome profiling of untreated and BBR treated human PBMCs derived from 4 PPD^+^ healthy individuals. Heat map representation of the differentially expressed proteins (Log2 fold, n = 3). Common proteins in all the individuals that are **(I)** downregulated and **(J)** upregulated upon BBR treatment. Biological processes that are **(K)** downregulated and **(L)** upregulated upon BBR treatment. **(M)** Notch signalling pathway associated proteins which were upregulated in human PBMCs upon BBR treatment. **(N)** RT-PCR of genes related to Notch signaling pathway. **(O)** Human CD4^+^ T cells expressing p-AKT and **(P)** human CD4^+^ T cells expressing p-FOXO1. **(Q)** MFI of human CD4^+^ T cells expressing Blimp-1. **(R)** Extracellular L-Lactate quantification in untreated and BBR treated human PBMCs. **(S-W)** represents multiplex cytokines assay upon BBR treatment in human PBMCs of derived from 4 PPD^+^ healthy individuals (refer methodology). **(S-V)** Fold expression changes of 46 cytokines upon BBR treatment in different individuals. **(W)** Heat map of common differentially expressed cytokines. In **H**, **I**, **J**, **M**, and **W**, Red represents upregulation while blue represents downregulation. Data is representative of two independent experiments. The data values represent mean ± SD (n is 4 to 7). *p<0.05, **p<0.005, ***p<0.0005.

**Fig 3A** represents a simplistic model of T cell differentiation into different memory subsets highlighting different regulators and surface markers used to identify these cells. To understand the molecular signaling involved in the enhancement of CD4^+^ adaptive memory after BBR treatment, we cultured PBMCs isolated from healthy PPD^+^ individuals in the presence of BBR for 48h. Intriguingly, BBR treatment drove significant differentiation of CD4^+^ T_NAIVE_ cells into T_EM_, T_EMRA_ and T_RM_ T cell subsets with no significant increase in T_CM_ cells **(Fig 3B–3G)**. To understand the mechanistic details of heightened memory responses, we performed the whole proteome analysis of human PBMCs with or without BBR treatment. In each individual, BBR treatment induced a distinct proteome landscape with a significant number of differentially expressed proteins **(Fig 3H)**. Interestingly, BBR treatment collectively downregulated the expression of 323 proteins **(Fig 3I)** and induced the expression of 527 proteins **(Fig 3J)** in the treated PBMCs. Further analysis on these commonly expressed proteins revealed few pathways which were downregulated upon BBR treatment **(Fig 3K)**. Curiously, diverse pathways associated with key cellular processes such as cell cycle, cell adhesion, cell division and glycolysis were upregulated upon BBR treatment **(Fig 3L)**. Importantly, BBR treatment enhanced the upregulation of critical proteins of Notch signaling pathway **(Fig 3L and 3M)** which is known to stimulate the differentiation and maintenance of T_RM_ cells [30].

TCR stimulation along with downstream signalling pathways such as PI3K/AKT/mTOR play a critical role in shaping the T cell memory [12]. While activated AKT is known to phosphorylate FOXO1 triggering its nuclear exclusion, previous literature highlights the importance of FOXO1 mediated gene expression in the generation and maintenance of protective memory cells [31,32] Further, Blimp1 transcription factor is known to regulate resident memory responses at the local site of infection [33,34]. Ironically, Notch3 transactivates PTEN which in turn inhibits the AKT signaling [35] leading to FOXO1 activation **(S7A Fig)**. We validated these targets by RT-PCR and observed that BBR treatment induced significant expression of genes related to notch signaling as well as the downstream targets such as Foxo1, Pten and Blimp1 **(Fig 3N)**. Moreover, BBR inhibited AKT and FOXO1 phosphorylation **(Figs 3O and 3P and S7B–S7D)** and along with AKT inhibitor (AKTi), BBR synergistically reduced T_NAIVE_ population **(S7E Fig),** increased T_EM_ **(S7F Fig)** and T_EMRA_ cells **(S7G Fig)** with no effect on T_CM_ cells **(S7H Fig)**. BBR treatment also enhanced resident memory functions by modulating the BLIMP-1 expression **(Fig 3Q)**.

Cellular metabolism plays a crucial role in modulating T cell effector functions [36] while BBR is known to induce glycolysis in many cell types [37,38], **(S7I Fig)**. Consistent with the previous findings and our proteomics data, BBR significantly induced glycolysis **(Fig 3R)** in human PBMCs. Furthermore, to dwell deeper into the immunological milieu induced upon BBR treatment, Bio-plex Pro Human cytokine screening Panel (48-Plex) was utilised to screen the regulation of cytokines in human PBMCs. BBR treatment consistently enhanced the expression of pro-inflammatory cytokines in human PBMCs **(Fig 3S–3V)** derived from PPD^+^ individuals. Moreover, 20 pro-inflammatory cytokines and chemokines were upregulated in the PBMCs derived from at least 3 out of 4 individuals **(Fig 3W)**. Overall these results indicate that BBR enhances effector functions of CD4^+^ T cells and upregulates critical signaling associated with T_RM_ establishment and maintenance.

### BBR drives the expansion of memory T cells by modulating key regulators of T cell development in murine T cells

**Fig 4:**
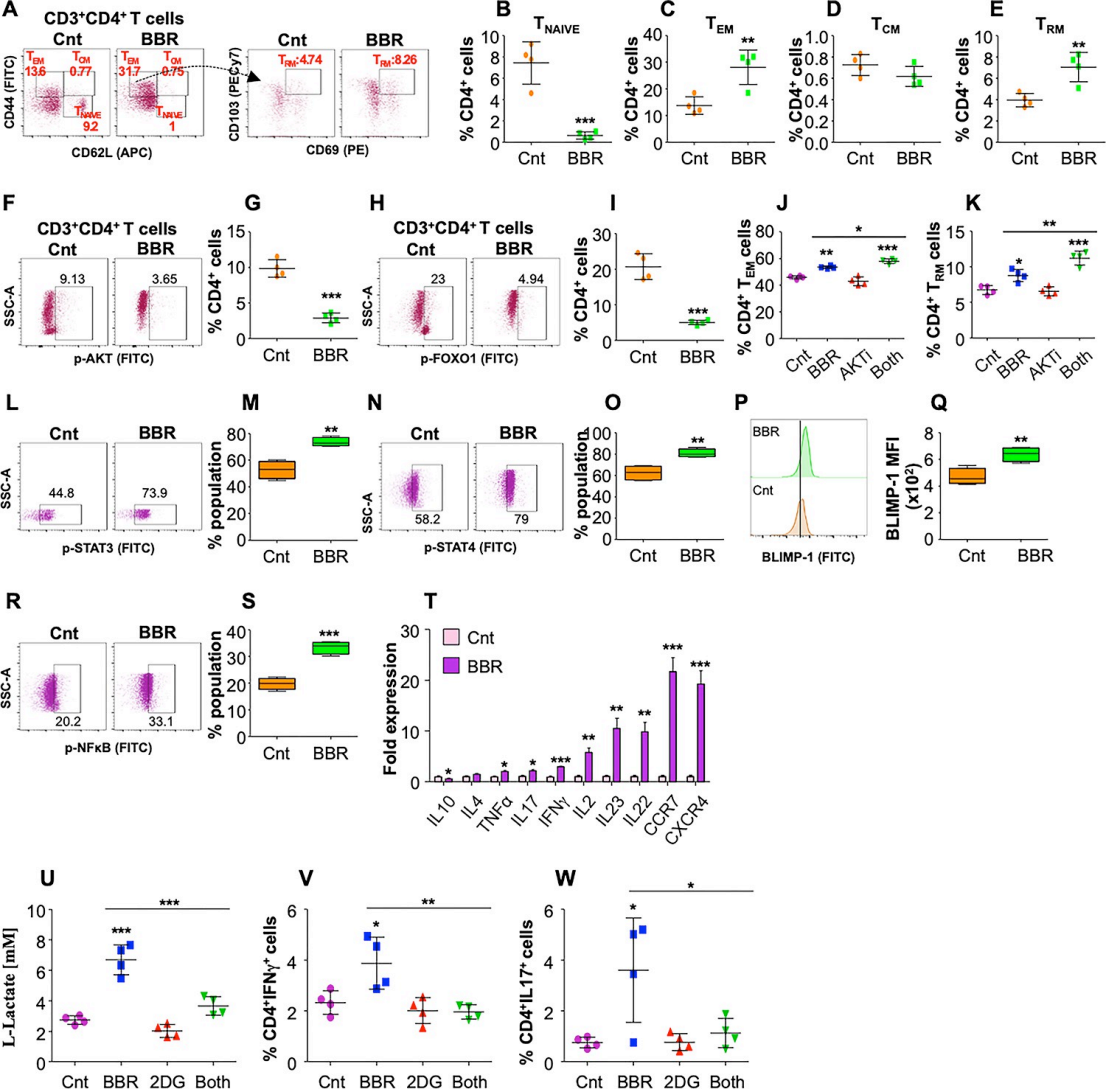
BBR induces expansion of antigen-specific memory T cells by targeting TCR signaling and glycolysis. Splenocytes isolated from *M*.*tb* infected mice were *ex vivo* stimulated with *M*.*tb* CSA and treated with BBR (10 μg/ml) for 48 h. *Ex vivo* stimulated splenocytes were surface stained with α-CD3 (Pacific Blue), α-CD4 (PerCPCy5.5), α-CD8 (APCCy7), α-CD69 (PE), α-CD103 (PECy7), α-CD62L (APC) and α-CD44 (FITC). **(A)** Representative dot plots and the percentage of **(B)** CD4^+^ T_NAIVE_ cells, **(C)** CD4^+^ T_EM_ cells, **(D)** CD4^+^ T_CM_ cells and **(E)** CD4^+^ T_RM_ cells after BBR treatment. **(F-I)** To analyse the activation status of key signaling molecules and transcription factors, the cells were stained with α-CD3 (Pacific Blue) and α-CD4 (PerCPCy5.5) followed by intracellular staining with antibodies against p-AKT and p-FOXO1 (see methods). **(F)** Representatives FACS scatter plots and **(G)** the percentage of CD4^+^ T cells expressing pAKT. **(H&I)** Representative scatter plots and the percentage of CD4^+^ T cells expressing p-FOXO1. **(J&K)** *Ex vivo* stimulated splenocytes were treated with BBR (10 μg/ml), AKTi (2.5μM) or both for 48 h followed by surface staining with α-CD3 (Pacific Blue), α-CD4 (PerCPCy5.5), α-CD69 (PE), α-CD103 (PECy7). **(J)** Percentage of CD4^+^ T_EM_ and **(K)** CD4^+^ T_RM_ cells. **(L-S)** Stimulation of transcription factors involved in memory responses were examined for which the cells were stained with α-CD3 (Pacific Blue) and α-CD4 (PerCPCy5.5) followed by intracellular staining with antibodies against p-STAT3, p-STAT4, BlIMP-1 and p-NFκB (see methods). Representatives FACS scatter plots and percentage of CD4^+^ T cells expressing **(L&M)** p-STAT3, **(N&O)** p-STAT4, **(P&Q)** Blimp-1 and **(R&S)** p-NFκB. **(T)** Expression of cytokines in *M*.*tb* specific T cells with or without BBR treatment. **(V-X)** *Ex vivo* stimulated splenocytes isolated from *M*.*tb* infected mice were treated with BBR (10 μg/ml), 2-Deoxy-D-glucose (2DG; 200 mM), both or left untreated for 24 h. **(U)** L-Lactate present in the supernatant of treated splenocytes. **(V)** Percentage of CD4^+^IFNγ^+^ T cells and **(W)** CD4^+^IL17^+^ T cells. The data values represent mean ± SD (n = 3–4). *p<0.05, **p<0.005, ***p<0.0005.

To validate the molecular signaling involved in the enhancement of CD4^+^ adaptive memory after BBR treatment, splenocytes isolated from *M*.*tb* infected mice were *ex vivo* stimulated with *M*.*tb* complete soluble antigen (CSA) and treated with BBR (10μg/ml) for 48h followed by immune profiling. Analysis of different CD4^+^ T cell subsets via flow cytometry **(Fig 4A)** revealed that BBR treatment significantly reduced the percentage of T_NAIVE_ subset **(Fig 4B)** with a concomitant increase in the T_EM_ cells **(Fig 4C)**. While no difference was observed in the T_CM_ population **(Fig 4D)**, BBR significantly induced the T_RM_ subset **(Fig 4E)**. Consistent with the previous results, BBR treatment significantly reduced the activation of AKT **(Fig 4F and 4G)** and decreased the phosphorylation of FOXO1 **(Fig 4H and 4I)** in CD4^+^ T cells. Inhibition of AKT signalling has been shown to promote central memory responses by increasing nuclear accumulation of FOXO1 [39]. It is also well-established that STAT3 and STAT4 play specific function in the formation of T cell memory subsets in response to infections [16,40]. To further ascertain the influence of BBR treatment on AKT-FOXO1 axis, we repeated the *ex vivo* T cell stimulation experiment in the presence of AKTi and BBR. AKTi alone did not increase the percentage of CD4^+^ T_EM_ and T_RM_ populations, whereas, considerable enrichment was observed upon BBR treatment alone or in combination with AKTi **(Fig 4J and 4K)**. This infers the influence of alternate signaling pathways contributing in enhancement of memory responses upon BBR treatment. We observed that BBR treatment induced the activation of STAT3, STAT4 and BLIMP-1 in CD4^+^ T cells **(Fig 4L–4Q)** which may lead to enhanced T_RM_ response. Interestingly, heightened NFκB activation was observed in BBR treated CD4^+^ T cells **(Fig 4R and 4S)** leading to a significant upregulation of host protective proinflammatory responses **(Fig 4T)**. Furthermore, BBR treatment led to enhanced glycolysis in CD4^+^ T cells **(Fig 4U)**. Interestingly, 2-Deoxy-D-glucose (2DG) (glycolysis inhibitor) treatment abrogated the expression of IFNγ and IL17 in BBR treated CD4^+^ T cells **(Fig 4V and 4W)** indicating that BBR potentiates pro-inflammatory response through metabolic reprogramming. Overall, this concludes that BBR treatment expands memory T cell subsets with proinflammatory characteristics.

### BBR induced adaptive memory enhances the BCG vaccine efficacy and reduces the rate of TB recurrence

**Fig 5:**
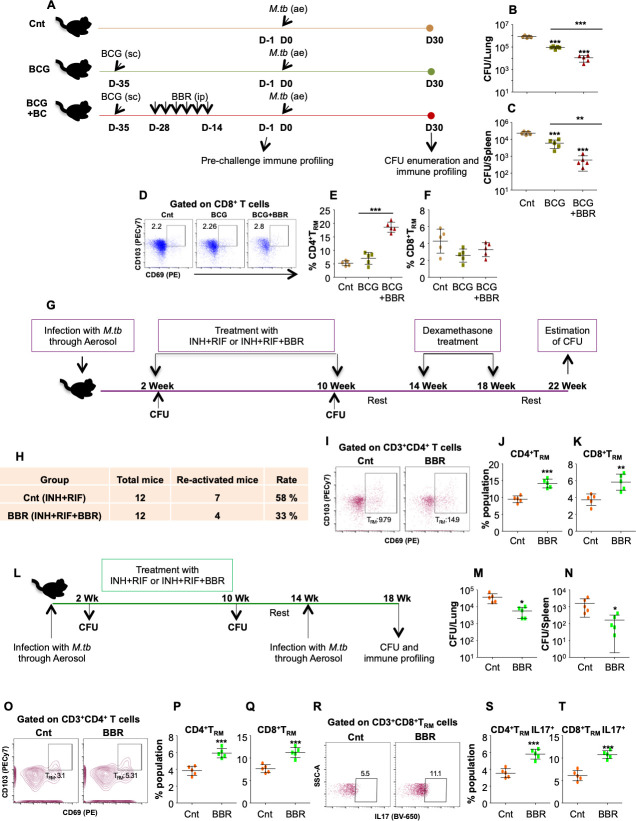
BBR enhances the BCG vaccine efficacy and protects against recurrent TB by inducing T cell resident memory responses in murine model. **(A)** Schematic representation of vaccine model used in the study. Bacterial load in **(B)** the lungs and **(C)** the spleen of infected mice. *Ex vivo* stimulated lung cells were surface stained with α-CD3 (Pacific Blue), α-CD4 (PerCPCy5.5), α-CD69 (PE), α-CD103 (PECy7), followed by flow cytometry. **(D)** Representative FACS plots and percentage of **(E)** CD4^+^ T_RM_ cells and **(F)** CD8^+^ T_RM_ cells in the lung of infected animals. **(G)** Schematic diagram representing the re-activation model used in this study. **(H)** Rate of disease relapse with and without BBR treatment. **(I)** FACS plots representing CD69 and CD103 expressing CD4^+^ T cells and **(J)** percentage of CD4^+^ T_RM_ cells and **(K)** CD8^+^ T_RM_ cells in the spleen of infected mice. **(L)** Diagrammatic representation of re-infection model used in the study. Bacterial burden in **(M)** the lungs and **(N)** the spleen of re-infected mice. *Ex vivo* stimulated single cell suspensions of the lungs were stained with α-CD3 (Pacific Blue), α-CD4 (PerCPCy5.5), α-CD69 (PE), α-CD103 (PECy7), α-CD62L (APC), and α-IL17 (BV650) followed by flow cytometry. **(O)** Representative FACS plots and the percentage of **(P)** CD4^+^ T_RM_ cells and **(Q)** CD8^+^ T_RM_ cells in the lungs of re-infected mice. **(R-T)** Percentage of resident memory T cells producing IL17. Data is representative of two independent experiments. The data values represent mean ± SD (n = 5). *p<0.05, **p<0.005, ***p<0.0005.

Having established the potential of BBR to induce significant immunological memory against TB, we next investigated whether BBR co-treatment could enhance the BCG vaccine efficacy *in vivo*. C57BL/6 mice were divided in 3 groups: Cnt (un-vaccinated), BCG (BCG vaccinated) and BCG-BBR (BCG vaccinated and BBR treated), and were challenged with low dose of H37Rv through aerosol infection model **(Fig 5A)**. 30 days after *M*.*tb* infection, the mice were euthanized and analysed for bacterial burden and immune profiling. Pre-challenge immune profiling of the animals revealed increased activation of CD4^+^ and CD8^+^ T cells in the lungs **(S8A–S8E Fig)** and the spleen **(S8F–S8I Fig)** of the BCG vaccinated and BBR treated animals as compared to the BCG vaccinated alone. Consistent with this, BBR treatment during BCG vaccination significantly decreased the bacterial burden in the lungs **(Fig 5B)** and the spleen **(Fig 5C)** of co-treated mice as compared to only BCG vaccination. Immune analysis revealed increased percentage of CD4^+^ and CD8^+^ T cells in the lungs **(S9A–S9C Fig)** as well as in the spleen **(S9D–S9F Fig)** of co-vaccinated mice. BCG primarily induces effector memory responses as a result of which the anti-TB immunity induced by BCG is short lived and wanes in adults [41]. Interestingly, with no effect in the lungs **(S9G–S9I Fig)**, BBR treatment increased the CD4^+^ and CD8^+^ T_CM_ cells in the infected spleen **(S9J–S9L Fig)**. Furthermore, the percentage of T_RM_ cells was significantly high in the lungs **(Fig 5E and 5F)** and the spleen **(S9M–S9O Fig)** of infected BCG-BBR vaccinated and treated animals as compared to the BCG vaccination alone.

Memory T cells are vital for long-term immunity against disease relapse due to re-activation or re-infection. To further provide the *in vivo* evidence of the above results, we performed reactivation study in murine model **(Fig 5G)**. BBR co-therapy significantly reduced the rate of disease re-activation **(Fig 5H)** further proving that BBR treatment generates long-term *M*.*tb* specific protective memory responses with boosted T_CM_ **(S10A–S10C Fig)** and T_RM_ populations **(Fig 5I–K)** in the lungs of infected mice. Furthermore, in re-infection murine model of TB **(Fig 5L)**, the bacterial burden was significantly reduced in the lungs **(Fig 5M)** and the spleen **(Fig 5N)** of re-infected mice previously treated with INH+RIF+BBR (BBR group) as compared with INH+RIF group (Cnt group). Immune profiling revealed increased percentage of T_RM_ cells in the lungs **(Fig 5O–5Q)** and the spleen **(S10D and S10E Fig)** of re-infected animals treated with INH+RIF+BBR. IL17 plays a crucial protective role in the recall protection to *M*.*tb* infection [6,11]. Interestingly, IL17 secreting T_RM_ population was enriched in the lungs **(Fig 5R–5T)** and the spleen **(S10F and S10G Fig)** of BBR treated mice. Similar trend was observed for the T_CM_ population in both the organs **(S10H–S10Q Fig)**. Collectively, our preclinical mice and human data projects BBR as an excellent immunotherapeutic and immunoprophylactic candidate against susceptible and drug resistant TB.

## Discussion

Immunological memory can be delineated as modification in immune responsiveness after the primary encounter to elicit prompt and robust immune responses. With more profound insights, the conventional characterization of immunological memory is continuously advancing. Since an upsurge in incidences of drug-resistant TB along with recurrence and reactivation of *M*.*tb* infection is the root cause of morbidity and mortality worldwide, the utmost significance of immunological memory to combat TB remains unchanged [42]. Therefore, an efficacious immunomodulatory strategy is deemed indispensable to enhance population-wide immune protection to moderate the global TB burden. Strategic host-directed therapies to augment protective immune responses, accomplish bacterial sterility, and subside detrimental pathology are endeavoured [24] to improve clinical outcomes in TB patients [43,44]. Combinatorial administration of immunotherapeutic has resolved inadequacies of numerous stratagems and was found advantageous in eliminating *M*.*tb* infections in several clinical trials [45]. One of the most established immunotherapeutic BBR has been known for ages for its effective therapeutic potential to treat diabetes and many other diseases [46,47]. Despite this, the prospects of BBR for better clinical recovery during *M*.*tb* infection by augmenting immunological memory responses have not been evaluated.

In line with the previous literature [27], in this study we demonstrated the anti-mycobacterial potential of BBR in murine macrophages, human monocytic cell line THP-1, and murine model against susceptible and drug-resistant strains of *M*.*tb*. Mononuclear phagocytic cells are prominent in activating T cells but during the pathogenic hijack, apoptotic inhibition occurs that restricts the presentation of bacterial antigen and further delays T cell mediated adaptive immune response [48]. We found that BBR prompted apoptotic cell death in infected macrophages which constrains *M*.*tb* infection. With little direct anti-mycobacterial activity, BBR greatly enhanced the host defence mechanisms by modulating NF-κB and STAT-3 signaling [49]. BBR also increased the percentage of co-stimulatory molecules on mouse peritoneal macrophages and enriched the host protective chemokines, cytokines in response to *M*.*tb* infection which was further trailed in line with T cell responses. BBR treatment also enhanced the killing potential of T cells as was evident by the results of *ex vivo* co-culture experiments. BBR administration in adjunct to INH reduced the bacterial burden in human monocytic cell line THP-1 as well as peritoneal macrophages. Furthermore, BBR significantly boosted the extermination of drug-resistant MDR and XDR strains of *M*.*tb*.

The therapeutic effects of BBR have been evaluated for diverse diseases in murine models, with no significant toxicity [50,51]. So, comprehensive immunological analysis was performed in the murine model of TB. Recently a piecemeal study has evaluated the impact of BBR treatment in adjunct to front-line TB drugs INH and RIF against drug-susceptible *M*.*tb* strain [27] wherein BBR as an adjunct did not demonstrate any additive or synergistic effect on the bacterial load. In our study, we have defied numerous shortcomings of this study. First of all, our study extensively validates the effectiveness of BBR in eliciting anti-mycobacterial immune responses against drug-susceptible and resistant strains of *M*.*tb* exclusive of ATT drugs. We also demonstrate the effectiveness of BBR in reversing the immune dampening effects of INH. Augmented efficacy of BBR in terms of lower bacterial burden, reduced lung inflammation and heightened host protective immune response either alone or in combination with INH can be attributable to a long treatment regimen of 45 days in our study. Moreover, BBR was administered intraperitoneally for superior circulation. Treatment was scientifically strategized to downgrade hepatotoxicity and to enhance host protective responses. BBR administration significantly reduced the bacterial load in adjunct to frontline anti-TB drug INH and resultant diminution in pathological damage in the lungs as reported recently [27]. Our results also demonstrate the effectiveness of BBR treatment in significantly lowering the bacterial load in MDR and XDR infected animals. Hence, it can be stated decisively that BBR elicits anti-mycobacterial immune responses against a range of *M*.*tb* strains. Furthermore, with no prior information on BBR induced mechanisms of protection against TB, we have comprehensively evaluated the impact of BBR on T cell signal transduction linking it with the establishment of durable immunological memory against *M*.*tb*.

To decipher the immunological feature of BBR induced reduction in bacterial burden, immune profiling was performed. Activation of adaptive immune cell populations was observed in compliance with an increase in the percentage and the activation of innate immune cells upon BBR treatment. In case of inflammatory bowel disease, BBR is known to induce protective immune responses by modulating IFNγ and IL17 CD4^+^ T cells by activation of AMP kinase [52]. IFNγ plays a vital role during *M*.*tb* infection by stimulating Th1 induction from naïve CD4^+^ T cells [53] and IL17 plays a significant role by stimulating recall responses during recurrent *M*.*tb* infections [11,54,55]. In agreement with the previous reports, BBR treatment resulted in the increased percentage of IFNγ and IL17 secreting CD4^+^ and CD8^+^ T cells. Furthermore, BBR treatment enhanced expression of pro-inflammatory cytokines such as CCL2, IL12β, IL1β alone and in adjunct to INH. Furthermore, INH induced anti-inflammatory responses were countered along with BBR treatment.

Long-term memory particularly increased T_CM_ pool is vital for heightened immune responses against *M*.*tb* infections. Further, the T_RM_ population residing at the site of infection play a critical role in mediating diverse host protective effector functions. T_RM_ upon encountering antigen stimulates IFNγ production and recruits memory T cell and other immune cell populations to the site of infection [56,57]. Our research presents strong evidence of the augmented T_RM_ and T_CM_ responses upon BBR therapy alone and in adjunct to INH. These results were further strengthened by BCG vaccination experiments wherein we observed a striking reduction in the bacterial burden on administering BBR post BCG immunization in *M*.*tb* infected mice. In agreement with previous results, we observed a superior percentage of T_CM_ and T_RM_ cell responses upon BBR treatment subsequent to BCG immunization. This again highlights the prominence of this immunomodulatory strategy to provide ever-lasting immunity against *M*.*tb* infections. Further, the biological evidence of long-term protective immune prophylactic effects of BBR were provided by re-infection and re-activation mice experiments wherein BBR treated animals displayed increased T_CM_ and T_RM_ cell responses leading to a significantly lower bacterial burden and reduced relapse rate.

To dwell into the immune mechanisms by which the attributes of immunological memory were enhanced upon BBR treatment, we performed *ex vivo* studies with *M*.*tb* specific T cells and observed that BBR treatment instigated significant differentiation of T_NAIVE_ population into T_EM_ and T_RM_ cells. It is well-established that naïve T cells, T_CM_ and T_EM_ cells are capable to differentiate into T_RM_ cells upon stimulation [58]. Previously it has been reported that the T_RM_ cells have a definite cytokine profile (TGFβ, IL15, Type I IFN, IL12) as well as T_RM-_specific transcription factors such as Runx3, Hobit, Blimp1etc [59] that distinguish them from other immune cell populations and enriching selective and protective tissue specific immunity. Apart from this, certain studies have ascertained the critical role of STAT4 in regulating T_RM_ differentiation and persistence in achieving tissue-specific immunity against infections [40]. *Ex vivo* BBR treatment elicited the expansion of T cell memory pool by modulating vital interconnected immune molecules such as AKT, FOXO-1, STAT-3, STAT-4, BLIMP-1 as well as NFκB. Instigation of memory establishment was in agreement with the enhancement of pro-inflammatory cytokines such as IFNγ, IL2, IL23, IL22, CCR7 and CXCR4 in BBR treated *M*.*tb* specific T cells. Furthermore, BBR induced metabolic flux towards glycolytic pathway thereby enhancing effector functions of CD4^+^ T cells secreting key host protective cytokines- IFNγ and IL17.

**Fig 6:**
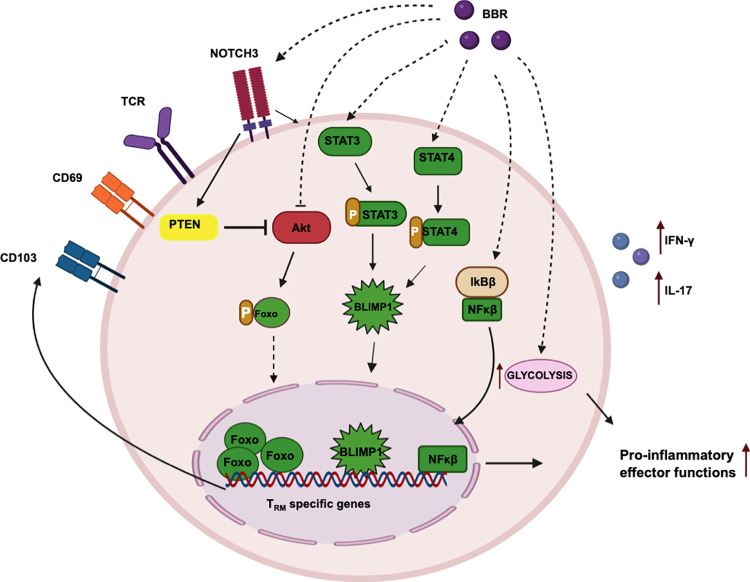
BBR instigates host-protective immune responses against M.tb by directing key immunological signaling pathways. In response to *M*.*tb* infection, BBR establishes long-lived, host protective resident memory T cells (TRM) at the site of infection. BBR enhances the effector functions of T lymphocytes by enhancing CD69 expression, directing metabolic flux towards glycolysis, activation of key host protective signaling pathways, and pro-inflammatory immune responses. BBR enriches pathways essential for the establishment and maintenance of memory T cells. BBR upregulates NOTCH3 which directs PTEN to simultaneously inhibit AKT and activate STAT signaling. AKT inhibition further decreases FOXO1 phosphorylation thereby enhancing its nuclear retention. BBR-mediated enhancement of activated STAT4 and STAT3-mediated BLIMP1 signaling axis further results in heightened expression of TRM-specific genes for long-term protection against M.tb infections.

Many times, the results obtained with mice studies poorly mimic conditions of human physiology. To ascertain the memory inducing potential of BBR in humans, we performed *ex vivo* experiments with PBMCs isolated from PPD^+^ healthy donors. To our satisfaction, BBR treatment resulted in a significant reduction in CD4^+^ T_NAIVE_ population with a concomitant increase in CD4^+^ T_EM_, T_EMRA_ and T_RM_ subsets. Moreover, BBR treated human PBMCs also displayed reduced phosphorylation of AKT which led to an enhanced FOXO1 activation as BBR/AKTi treatment synergistically decreased CD4^+^ T_NAIVE_ population and increased T_EM_ and T_EMRA_ subsets. Additionally, BLIMP-1, a T_RM_ specific transcription factor was also upregulated in the BBR treated PBMCs [60]. Similar to the mice *ex vivo* experiments, BBR treatment heightened glycolytic flux in human PBMCs along with boosted pro-inflammatory cytokine profile. Furthermore, whole proteome analysis revealed upregulation of vital cellular processes in BBR treated human PBMCs including maintenance of glycolysis which is necessitated for T cell effector functions [61] and Notch signaling pathway critically known for T_RM_ establishment. Receptors, NOTCH1/3 and activators APHA1/PSEN were found to be upregulated at the RNA as well as the protein levels in the BBR treated hPMBCs. Notch signaling is known to induce PTEN expression which in turn activates FOXO1 by inhibiting AKT [35,62]. Convincingly, BBR treatment significantly induced the expression of PTEN and FOXO1 in human PMBCs. Based on these results; we propose a probable mechanism of action by which BBR enhances the immunological memory against TB **(Fig 6)**.

Finally, this study substantiates the prospects of BBR as a potent immunomodulator that strikingly augments protective immunological memory responses against *M*.*tb* infection. Further corroboration in the higher TB model that shares more similarities with humans such as non-human primates will be valuable to appraise BBR as promising host-directed therapy against susceptible and drug-resistant TB.

## Methodology

### Ethics statement

Animal experiments were executed as per the regulations stated by the Institutional Animal Ethics Committee of the International Centre for Genetic Engineering and Biotechnology (ICGEB, New Delhi, India) along with the Department of Biotechnology (DBT) standards (Government of India) (Approval ID: ICGEB/IAEC/08/2016/IMB-1, ICGEB/IAEC/08092021/IMB-19). All the animals utilized in the investigation were ethically sacrificed by asphyxiation with carbon dioxide adhering to institutional and DBT practices.

All the experiments related to human samples were performed as per the regulations stated by the Institutional Ethics Committee of Institute of Liver and Biliary Sciences (ILBS), New Delhi along with the Department of Biotechnology (DBT) standards (Government of India) (Approval ID:IEC/2021/70/NA6). Human samples were handled by a skilled technician, samples were coded to maintain records, and consent forms were signed by all the participants before sample acquisition.

### Mice

C57BL/6 mice of 6–8 weeks were maintained in the Animal facility at ICGEB, New Delhi, India. Mice were accessed and obtained for experimental procedures from the facility.

### Bacteria

The mycobacterial strains used in this study (H37Rv, Rv-GFP, MDR, and XDR) were maintained in 7H9 (Middlebrook, Difco) medium supplemented with 10% ADC (albumin, dextrose, and catalase; Difco), 0.05% Tween 80 and 0.2% glycerol. Axenic mid-log phase cultures were cryopreserved in 20% glycerol (Sigma) and were kept at -80°C for future purpose.

### Peritoneal macrophages

Mice were intraperitoneally injected with 2ml of 4% thioglycolate broth (BD) five days prior to the experiment. Cells were isolated from the peritoneal cavity using ice-cold sterile PBS and were cultured in RPMI-1640 medium supplemented with 10% fetal bovine serum (Thermo fisher scientific Inc or Hyclone) followed by overnight incubation at 37°C and 5% CO_2_. Non-adherent cells were removed by washing with sterile PBS while adherent monolayer of cells was used for further experiments. The homogeneity of isolated macrophages was analysed by staining with CD11b antibody followed by flow cytometry.

### *Ex vivo* infection, cell death, Apoptosis assay & CellROX assay

Peritoneal macrophages or PMA- differentiated THP1 cells were used for *ex vivo* experiments. For checking the cytotoxicity of BBR, mouse peritoneal macrophages were treated with different concentrations of BBR for 48 h followed by propidium iodide (PI) staining as described elsewhere [21]. Since 20 μg/ml of BBR was chosen for future experiments, a time dependent PI cytotoxicity assay was performed at this concentration. For infections, bacterial cryostocks were revived and single cell suspensions were formed. Macrophages were infected with *M*.*tb* strains at 1:10 MOI. Four hours post-infection, cells were washed twice with 1X PBS so as to remove the extracellular bacteria. Cells were treated with 20 μg/ml of BBR and then incubated at 37°C for different time points followed by CFU enumeration [63], immunoblotting, RNA isolation or flow cytometry. Apoptosis was detected by staining the cells with Annexin V/7AAD (Biolegend) as per manufacturer’s protocol. CellROX (ThermoFisher Scientific) was used to detect the intracellular ROS in the *M*.*tb* infected peritoneal macrophages after treatment with 20 μg/ml of BBR for 24 h [64].

### Human PBMC isolation

Blood samples from 10 healthy individuals were collected in BD vacutainer blood collection tubes and were layered onto Histopaque 1077 (Sigma-Aldrich) followed by centrifugation at 400g for 30 minutes at 25°C. The opaque interface containing PBMCs were gently transferred and suspended in complete RPMI-1640 medium. These cells were then pelleted, counted, and seeded in the 12-well plates for further experiments.

### Western blot analysis

RIPA buffer (50 mM Tris, pH 8.0, 150 mM NaCl, 1.0% NP-40, 0.5% Sodium deoxycholate, 0.1% SDS) supplemented with 1X protease and phosphatase inhibitor cocktail (thermos scientific) was used to lyse the cells. Protein concentration in samples was estimated using Bradford assay. Samples were run on 10% polyacrylamide gel followed by transfer on PVDF membrane (Millipore). Membrane was then blocked with 5% BSA dissolved in PBST (PBS and 0.1% Tween-20), followed by overnight probing for diverse proteins with respective antibodies. Chemiluminescent HRP substrate (ECL, Millipore) was layered to develop blots on ImageQuant LAS 500.

### qPCR analysis

Total RNA was isolated from peritoneal macrophages and splenocytes by standard RNA isolation protocol followed by cDNA synthesis using iScript cDNA synthesis kit (Bio-Rad). Real-time PCR was performed using SYBR Green Master Mix (Bio-Rad). Bio-Rad Real-Time thermal cycler (BioRad, USA) was used for Real-time quantitative RT-PCR analysis. The list of Primers used in the study are provided in the S1 Table.

### Mice infection with *M*.*tb* and CFU enumeration

Mice were infected with drug-susceptible and resistant strains of *M*.*tb* via aerosol route using Madison aerosol chamber (University of Wisconsin, Madison, WI) with its nebulizer pre-adjusted to deposit approximately 110 CFUs to the lungs of mice. Axenic cultures were sonicated to prepare 15 ml of bacterial single-cell suspension for infection. 5–6 mice from each group were euthanized at different time points to determine the bacterial burden. Lungs and spleen were isolated and homogenized in sterile PBS and were plated onto 7H11 Middlebrooks (Difco) plates containing 0.05% Tween-80, 10% oleic acid, albumin, dextrose, and catalase (OADC) (Difco). The homogenates were plated in different dilutions and were incubated at 37°C for 21–28 days. *M*.*tb* colonies were counted and CFU was enumerated at various time points.

### Drug administration

For *ex vivo* infection experiments, macrophages were treated with 20μg/ml of BBR (Sigma). Furthermore, immunological memory was analysed in human PBMCs and mice splenocytes utilising 10 μg/ml of BBR (Sigma). For mice studies, 4 mg/kg of BBR dissolved in 100 μl of PBS with 5% DMSO was injected intraperitoneally for 45 days thrice a week, whereas the control group received only vehicle. 100 mg/L of INH and 60 mg/L RIF were administered in drinking water which was changed every alternative day.

### Adoptive cell transfer therapy

To ascertain the *M*.*tb* specific immune response, the harvested lungs from BBR treated group was macerated using sterile frosted slides to prepare single cell suspension. Subsequently the stained CD4^+^ T cells were sorted and cultured overnight in complete RPMI. Approximately one million cells were injected intravenously to Rag1-/- mice and further given low dose aerosol 5 days post transfer. Lung and spleen were then isolated after 21 days to determine the bacterial load.

### Multiplex cytokines immunoassay

Supernatant of 24h cultured Human PBMCs was collected and dilutions were prepared for sample and standard using Bio-Plex Pro Human Cytokines Assay Instructions manual. Detecting antibody was further added followed by Streptavidin-PE incubation. Data was acquired using Bio-Plex system and then analysed.

### Mass spectroscopy

10μg of protein was isolated from Control and BBR treated Human PBMCs and then desalted by reduction, alkylation and digestion using Trypsin Gold, Mass Spectrometry Grade (Promega Corporation, WA, USA) for 24hrs at 37°C for LC-MS/MS analysis. The peptides formed were extracted on a 25-cm analytical C18 column (C18, 3 μm, 100 Å), by (5–95%) gradient of buffer B (aqueous 80% acetonitrile and 0.1% formic acid) at a flow rate of 300 nL/min for 2.5 hrs. Subsequently, these peptides were exposed to nano-electrospray ionisation and Tandem mass spectrometry (MS/MS) by the application of Q-Exactive (Thermo Fisher Scientific, San Jose, CA, United States) at the collision-induced dissociation mode with the electrospray voltage of 2.3 kV. Data analysis comprising of standard statistical analysis, network and pathway analysis was done by Proteome Discoverer (version 2.0, Thermo Fisher Scientific, Waltham, MA, United States).

### BCG vaccination

Mice were vaccinated subcutaneously with the single dosage of 1 × 10^6^ colony forming units (CFUs) of BCG Pasteur strain in 100 μL of sterile saline. Subsequent to 7 days rest post-vaccination, the mice were treated with 4 mg/kg of BBR, thrice a week for 14 days intraperitoneally. After 14 days of break, the mice were then challenged via aerosol infection of *M*.*tb* strain H37Rv. Organs were harvested to elucidate bacterial burden and immune profiling within 30 days post-infection.

### Reinfection and Reactivation experiments

To undermine the susceptibility of *M*.*tb* infection, studies were performed in reactivation and reinfection models. Low dose aerosol of H37Rv was given to the mice and treated with INH (100 mg/L) and RIF (40 mg/kg) in drinking water for 12 weeks and rested for 30 days. Thereafter, the group was again challenged with *M*.*tb* infection followed by immune response and bacterial load determination. For reactivation studies, the mice were given dexamethasone (5 mg/kg) intraperitoneally, thrice a week for 30 days and so enumerated CFU and host protective immunological profiles.

### L-Lactate quantification

Extracellular L-Lactate levels were measured in the culture supernatant from splenocytes treated with BBR and 2DG. The experiment was performed using L- Lactate assay kit (Cayman Chemicals) as per the manufacturer’s guidelines.

### Flow cytometry

Lungs and spleen from mice of different groups were isolated and macerated using frosted slides in ice-cold RPMI 1640 (Hyclone) supplemented with 10% FBS to prepare single-cell suspension. RBC lysis buffer was used to lyse RBCs and cells were washed with 10% RPMI 1640. After cell counting, 1×10^6^ cells were seeded in 12 well for staining. For surface staining, cells were activated by 10 μg/mL of H37Rv complete soluble antigen (CSA) stimulation. Subsequently, 0.5 μg/mL Brefeldin A and 0.5 μg/mL of Monensin solution (BioLegend) were added during the last 4 hours of culture. Cells were then washed twice with FACS buffer (PBS + 3% FBS) and stained with antibodies directed against surface markers followed by fixation with 100 μl fixation buffer (biolegend) for 30 min. For intracellular staining, the cells were permeabilized using 1X permeabilizing buffer (Biolegend) and then were stained with fluorescently labelled anti-cytokine antibodies. For non-flurochrome tagged antibodies, secondary antibody tagged with Alexa Fluor 488 was used to measure the flurochrome intensity. The intensity of fluorochromes were assessed by flow cytometry (BD LSRFortessa Cell Analyzer—Flow Cytometers, BD Biosciences) followed by data analysis via FlowJo (Tree Star, USA).

### Antibodies

Following antibodies were used for this study:

Anti-Mouse: CD3-Pacific Blue, CD4-PerCPCy5.5, CD8-APCCy7, CD69-PE, CD44-FITC, CD62L-APC, CD103-PeCy7, CD69-FITC, IFNγ-APC, IFNγ-BV510, IL17-PECy7, IL17-BV650, CD11b-APCCy7, CD11c-APC, CD80-FITC, CD86-PerCPCy5.5, CD40-PE, CD4-PE, CD4-APC and CD4-FITC, CD3-BV510, CD3-BV650 from Biolegend, USA.

Anti-human: CD3-PE, CD4-APCCy7, CD8-Pacific Blue, CD45RO (PerCPCY5.5), CCR7 (PeCy7), CD69 (Alexa Fluor 700) from BD Biosciences.

Anti-human/anti-mouse: STAT3, p-STAT3, STAT4, p-STAT4, AKT, p-AKT, FOXO1, p-FOXO1, NFκB, p-NFκB, BLIMP1 and β-Tubulin from Cell Signaling Technology.

### Histopathology

Lungs harvested from infected animals were fixed with 10% neutral buffered formalin, and H&E staining was performed on 5-μm-thick paraffin-embedded tissues and were examined under microscope. Granulomas for each animal in every group were screened in 5 different fields. Submitted images are representative of all the visualized section images.

### Statistical analysis

All the experimental data was analysed using GraphPad Prism Software. Significant differences between the groups were determined by 2 tailed unpaired Student’s t-test or 1-way ANOVA. Human data was analysed by 2 tailed paired Student’s t-test. *p < 0.05, **p < 0.005, ***p< 0.0005.

## Supporting information

S1 Fig BBR possess weak anti-mycobacterial activity. Exponential cultures of *M*.*tb* strains H37Rv, MDR (Jal 2261) and XDR (MYC 431) were treated with different concentrations of BBR. OD_600_ of **(A)** H37Rv, **(B)** MDR, and **(C)** XDR cultures at day 0, day 2 and day 5 post treatment with BBR. **(D)** Cytotoxicity of BBR (different concentrations) on mouse peritoneal macrophages determined by PI staining at 48h post treatment. **(E)** Time kinetics of cytotoxicity of BBR (20 μg/ml) on mouse peritoneal macrophages determined by PI staining. **(F)** Representative histograms and **(G)** quantification of cellular ROS in *M*.*tb* infected macrophages with and without BBR treatment. **(H)** Percentage of apoptotic cells in uninfected macrophages 48h after treatment with BBR (20μg/ml). **(I)** OD_600_ of H37Rv cultures treated with BBR (20μg/ml) or INH (1μg/ml) or both for 5 days. Data is representative of at least two independent experiments. The data values represent mean ± SD (n = 3 to 4). *p<0.05, **p<0.005, ***p<0.0005.
(TIFF)

S2 Fig BBR activates innate immune cells in the lungs and the spleen of infected mice. **(A-H)** Single cell suspensions generated from the infected lungs and spleen were *ex vivo* stimulated with *M*.*tb* complete soluble antigen (CSA) for 16 h followed by surface staining with antibodies against CD11b (APCCy7), CD11c (APC), CD80 (FITC) and CD86 (PerCPCy5.5) and subjected to flow cytometry. **(A)** Representative contour plots and the percentage of **(B)** CD11b^+^ and **(C)** CD11c^+^ cells in the infected lungs. **(D)** Representative dot plots and the percentage of **(E)** CD11b^+^CD80^+^, **(F)** CD11b^+^CD86^+^, **(G)** CD11c^+^CD80^+^ and **(H)** CD11c^+^CD86^+^ cells in the lungs of infected animals. **(I)** Representative dot plots depicting the percentage of **(J)** CD11b^+^ and **(K)** CD11c^+^ cells in the infected spleen. **(L)** Representative dot plots and the percentage of **(M)** CD11b^+^CD80^+^, **(N)** CD11b^+^CD86^+^, **(O)** CD11c^+^CD80^+^, and **(P)** CD11c^+^CD86^+^ in the infected spleen. Data is representative of two independent experiments. The data values represent mean ± SD (n = 5). *p<0.05, **p<0.005, ***p<0.0005.
(TIFF)

S3 Fig BBR induces the activation of Th1/ Th17 immune responses in the spleen of infected mice. (**A-E)** *Ex vivo* stimulated splenocytes were surface stained with α-CD3 (Pacific Blue), α-CD4 (PerCPCy5.5), α-CD8 (APCCy7) and α-CD69 (FITC) followed by flow cytometry. **(A)** FACS dot plots and the percentage of **(B)** CD4^+^, **(C)** CD4^+^CD69^+^, **(D)** CD8^+^ and **(E)** CD8^+^CD69^+^ T cells in the spleen of infected mice. **(F-J)** *Ex vivo* stimulated splenocytes treated with monensin and brefeldin A for 2h and surface stained with α-CD3 (Pacific Blue), α-CD4 (PerCPCy5.5) and α-CD8 (APCCy7) followed by intracellular staining with α-IFNγ (APC) and α-IL17 (PECy7). **(F)** Representative dot plots and the percentage of **(G)** CD4^+^IFNγ^+^, **(H)** CD4^+^IL17^+^, **(I)** CD8^+^INFγ^+^ and **(J)** CD8^+^IL17^+^ cells in the infected spleen. Data is representative of two independent experiments. The data values represent mean ± SD (n = 5). *p<0.05, **p<0.005, ***p<0.0005.
(TIFF)

S4 Fig Memory T cell responses in the spleen of infected mice. *Ex vivo* stimulated splenocytes were surface stained with α-CD3 (Pacific Blue), α-CD4 (PerCPCy5.5), α-CD8 (APCCy7), α-CD62L (APC) and α-CD44 (FITC) followed by flow cytometry. **(A)** Representative dot plots and the percentage **of (B)** CD4^+^ T_CM_ cells and **(C)** CD8^+^ T_CM_ cells in the infected spleen. **(D-F)** T^RM^ cells were analysed by staining the splenocytes with α-CD3 (Pacific Blue), α-CD4 (PerCPCy5.5), α-CD8 (APCCy7), α-CD69 (FITC) and α-CD103 (APC) followed by flow cytometry. **(D)** Representative scatter dot-plot images and the percentage of **(E)** CD4^+^ T_RM_ cells and **(F)** CD8^+^ T_RM_ cells in the spleen of infected mice. Data is representative of two independent experiments. The data values represent mean ± SD (n = 5). *p<0.05, **p<0.005, ***p<0.0005.
(TIFF)

S5 Fig BBR induces adaptive immune responses against drug-resistant TB. **(A-H)** *Ex vivo* stimulated lung cells isolated from the mice infected with MDR and XDR *M*.*tb* were surface stained with antibodies against CD3 (Pacific Blue), CD4 (PerCPCy5.5), CD8 (APCCy7) and CD69 (FITC) followed by flow cytometry. Percentage of CD4^+^, CD4^+^CD69^+^, CD8^+^, CD8^+^CD69^+^ T cells in the lungs of mice infected with **(A-D)** MDR TB and **(E-H)** XDR TB. **(I-P)** Stimulated lung cells were treated with monensin and brefeldin A followed by surface staining with α-CD3 (Pacific Blue), α-CD4 (PerCPCy5.5) and α-CD8 (APCCy7) and intracellular staining with α-IFNγ (APC) and α-IL17 (PECy7). Percentage of CD4^+^INFγ^+^, CD4^+^IL17^+^, CD8^+^IFNγ^+^, CD8^+^IL17+ T cells in the lungs of mice infected with **(I-L)** MDR and **(M-P)** XDR strains of *M*.*tb*. Data is representative of two independent experiments. The data values represent mean ± SD (n = 5). *p<0.05, **p<0.005, ***p<0.0005.
(TIFF)

S6 Fig BBR activates Th1/Th17 immune responses in the spleen of mice infected with MDR/XDR TB. *Ex vivo* stimulated splenocytes isolated from MDR and XDR infected mice were analysed for T cell responses as described earlier. Percentage of CD4^+^, CD4^+^CD69^+^, CD8^+^ and CD8^+^CD69^+^ T cells in the spleen of mice infected with **(A-D)** MDR and **(E-H)** XDR strains of *M*.*tb*. Percentage of CD4^+^INFγ^+^, CD4^+^IL17^+^, CD8^+^IFNγ^+^ and CD8^+^IL17^+^ T cells in the spleen of mice infected with **(I-L)** MDR and **(M-P)** XDR strains of *M*.*tb*. Data is representative of two independent experiments. The data values represent mean ± SD (n = 5). *p<0.05, **p<0.005, ***p<0.0005.
(TIFF)

S7 Fig BBR induces T cell memory by modulating AKT-FOXO1 signaling in human CD4^+^ T cells. **(A)** Flowchart depicting the critical components involved in the transcription of T cell resident memory-specific genes. FACS plots from a single donor representing the percentage of CD4^+^ T cells expressing **(B)** p-AKT and **(C)** p-FOXO1. **(D)** Representative FACS scatter plots and the percentage of **(E)** CD4^+^ T_NAIVE_ cells, **(F)** CD4^+^ T_EM_ cells, **(G)** CD4^+^ T_EMRA_ cells and **(H)** CD4^+^ T_CM_ cells in the human PBMCs treated with BBR and AKTi. **(I)** Proposed model of enhanced T cell effector functions upon BBR treatment. The data values represent mean ± SD (n is 7). *p<0.05, **p<0.005, ***p<0.0005.
(TIFF)

S8 Fig Pre-challenge immune status of vaccinated animals. The lungs and the spleen of control, BCG and BCG-BBR vaccinated animals were harvested and analysed for T cell activation as described before. **(A)** Representative FACS plots and scatter plots depicting the percentage of CD4^+^, CD4^+^CD69^+^, CD8^+^ and CD8^+^CD69^+^ T cells in **(B-E)** the lungs and **(F-I)** the spleen of vaccinated animals before *M*.*tb* challenge. Data is representative of two independent experiments. The data values represent mean ± SD (n = 4). *p<0.05, **p<0.005, ***p<0.0005.
(TIFF)

S9 Fig BBR elevates BCG induced adaptive memory responses in the lungs and the spleen of infected mice. Percentage of CD4^+^ and CD8^+^ T cells in **(A-C)** the lungs and **(D-F)** the spleen of infected animals. Percentage of CD4^+^ T_CM_ cells and CD8^+^ T_CM_ cells in **(G-I)** the lungs and **(J-L)** the spleen of infected animals. **(M)** FACS plots and quantification of **(N)** CD4^+^ T_RM_ cells and **(O)** CD8^+^ T_RM_ cells in the spleen of infected animals. Data is representative of two independent experiments. The data values represent mean ± SD (n = 5). *p<0.05, **p<0.005, ***p<0.0005.
(TIFF)

S10 Fig Berberine counteracts recurrent TB by inducing IL17 producing memory T cells. **(A)** FACS plots representing the percentage of **(B)** CD4^+^ T_CM_ cells and **(C)** CD8^+^ T_CM_ cells in the lungs of re-activation group mice. Percentage of **(D)** CD4^+^ T_RM_ cells, **(E)** CD8^+^ T_RM_ cells, **(F)** CD4^+^IL17^+^ T_RM_ cells and **(G)** CD8^+^IL17^+^ T_RM_ cells in the spleen of re-infected mice. **(H)** FACS plots representation and the percentage of CD4^+^ T_CM_ cells and CD8^+^ T^CM^ cells in **(I&J)** the spleen and **(K&L)** the lungs of re-infected mice. **(M-Q)** Percentage of central memory T cells producing IL17. **(M)** Representative FACS scatter plots of and the percentage ofCD4^+^IL17^+^ T_CM_ cells andCD8^+^IL17^+^ T_CM_ cells in **(N&O)** the spleen and **(P&Q)** the lungs of re-infected mice. Data is representative of two independent experiments. The data values represent mean ± SD (n is 5). *p<0.05, **p<0.005, ***p<0.0005.
(TIFF)

S1 Table List of the Primers used in the study. (DOCX)
