# Supplementary figures and images for: Berberine governs NOTCH3/AKT signaling to enrich lung-resident memory T cells during tuberculosis
Source: PLoS Pathog. 2023 Mar 7;19(3):e1011165. doi: 10.1371/journal.ppat.1011165 (PMC9990925; doi:10.1371/journal.ppat.1011165)

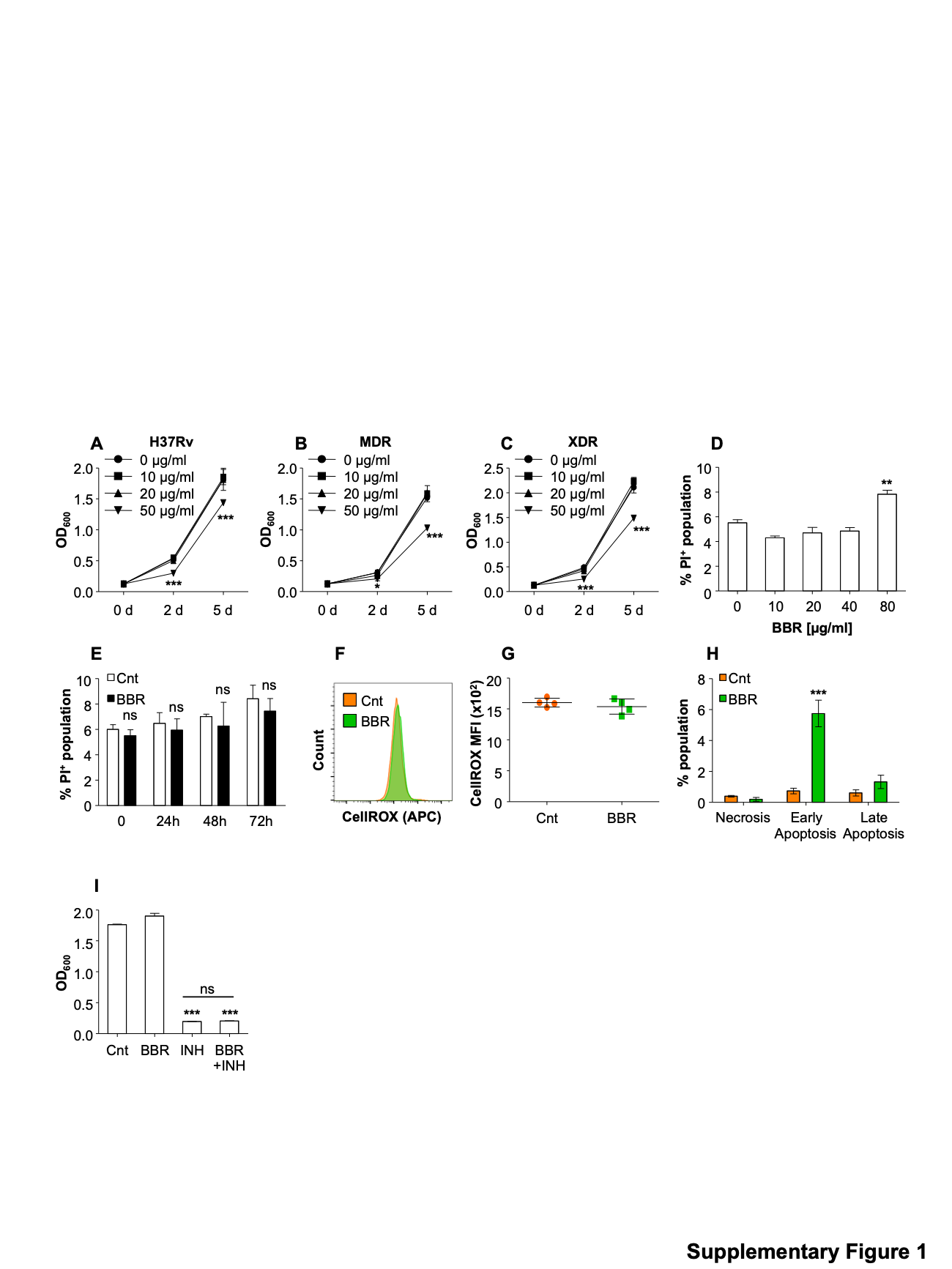

Supplement: S1 Fig — Exponential cultures of M.tb strains H37Rv, MDR (Jal 2261) and XDR (MYC 431) were treated with different concentrations of BBR. OD600 of (A) H37Rv, (B) MDR, and (C) XDR cultures at day 0, day 2 and day 5 post treatment with BBR. (D) Cytotoxicity of BBR (different concentrations) on mouse peritoneal macrophages determined by PI staining at 48h post treatment. (E) Time kinetics of cytotoxicity of BBR (20 μg/ml) on mouse peritoneal macrophages determined by PI staining. (F) Representative histograms and (G) quantification of cellular ROS in M.tb infected macrophages with and without BBR treatment. (H) Percentage of apoptotic cells in uninfected macrophages 48h after treatment with BBR (20μg/ml). (I) OD600 of H37Rv cultures treated with BBR (20μg/ml) or INH (1μg/ml) or both for 5 days. Data is representative of at least two independent experiments. The data values represent mean ± SD (n = 3 to 4). *p<0.05, **p<0.005, ***p<0.0005. (TIFF) [file ppat.1011165.s001.tiff]

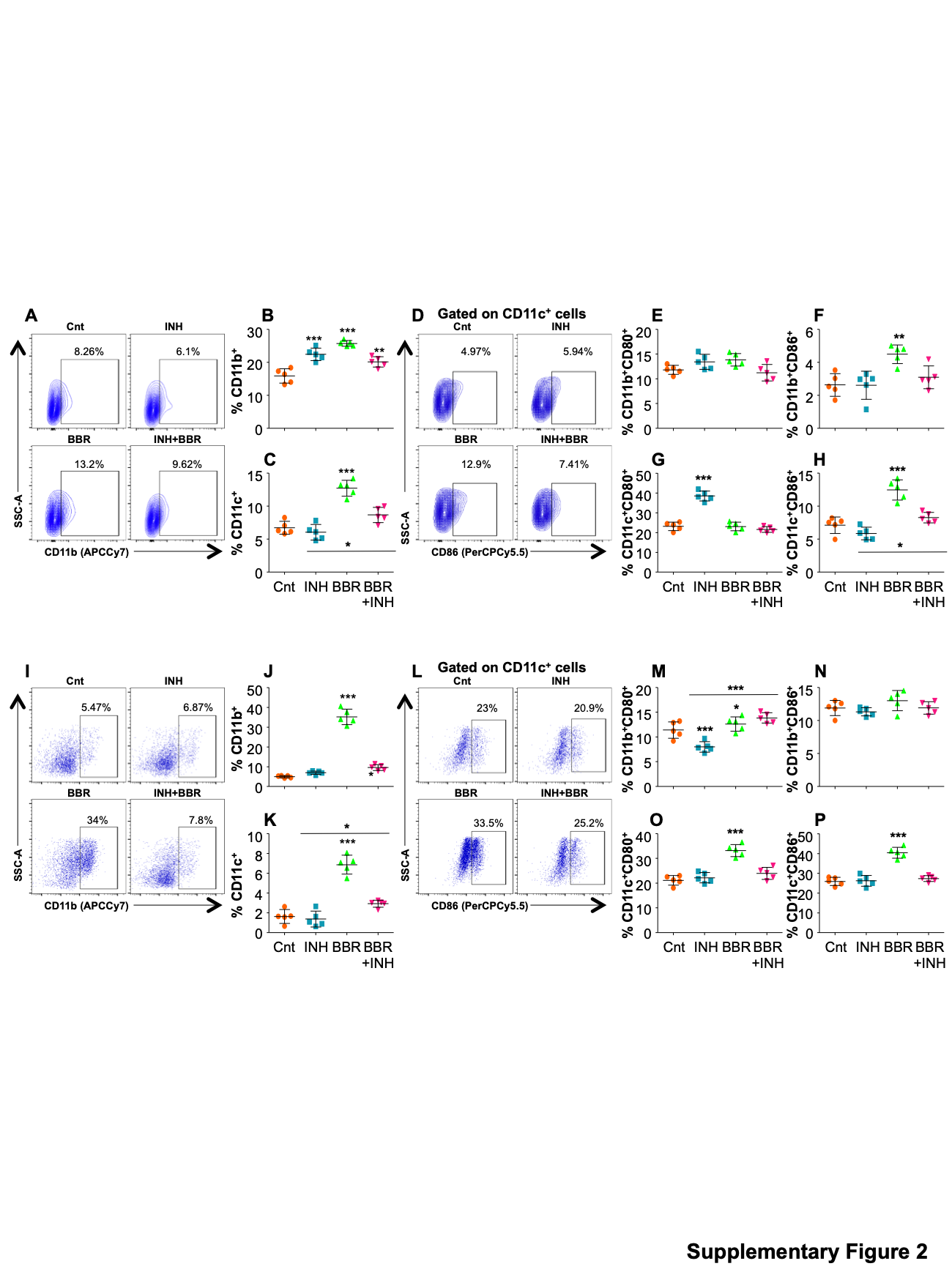

Supplement: S2 Fig — (A-H) Single cell suspensions generated from the infected lungs and spleen were ex vivo stimulated with M.tb complete soluble antigen (CSA) for 16 h followed by surface staining with antibodies against CD11b (APCCy7), CD11c (APC), CD80 (FITC) and CD86 (PerCPCy5.5) and subjected to flow cytometry. (A) Representative contour plots and the percentage of (B) CD11b+ and (C) CD11c+ cells in the infected lungs. (D) Representative dot plots and the percentage of (E) CD11b+CD80+, (F) CD11b+CD86+, (G) CD11c+CD80+ and (H) CD11c+CD86+ cells in the lungs of infected animals. (I) Representative dot plots depicting the percentage of (J) CD11b+ and (K) CD11c+ cells in the infected spleen. (L) Representative dot plots and the percentage of (M) CD11b+CD80+, (N) CD11b+CD86+, (O) CD11c+CD80+, and (P) CD11c+CD86+ in the infected spleen. Data is representative of two independent experiments. The data values represent mean ± SD (n = 5). *p<0.05, **p<0.005, ***p<0.0005. (TIFF) [file ppat.1011165.s002.tiff]

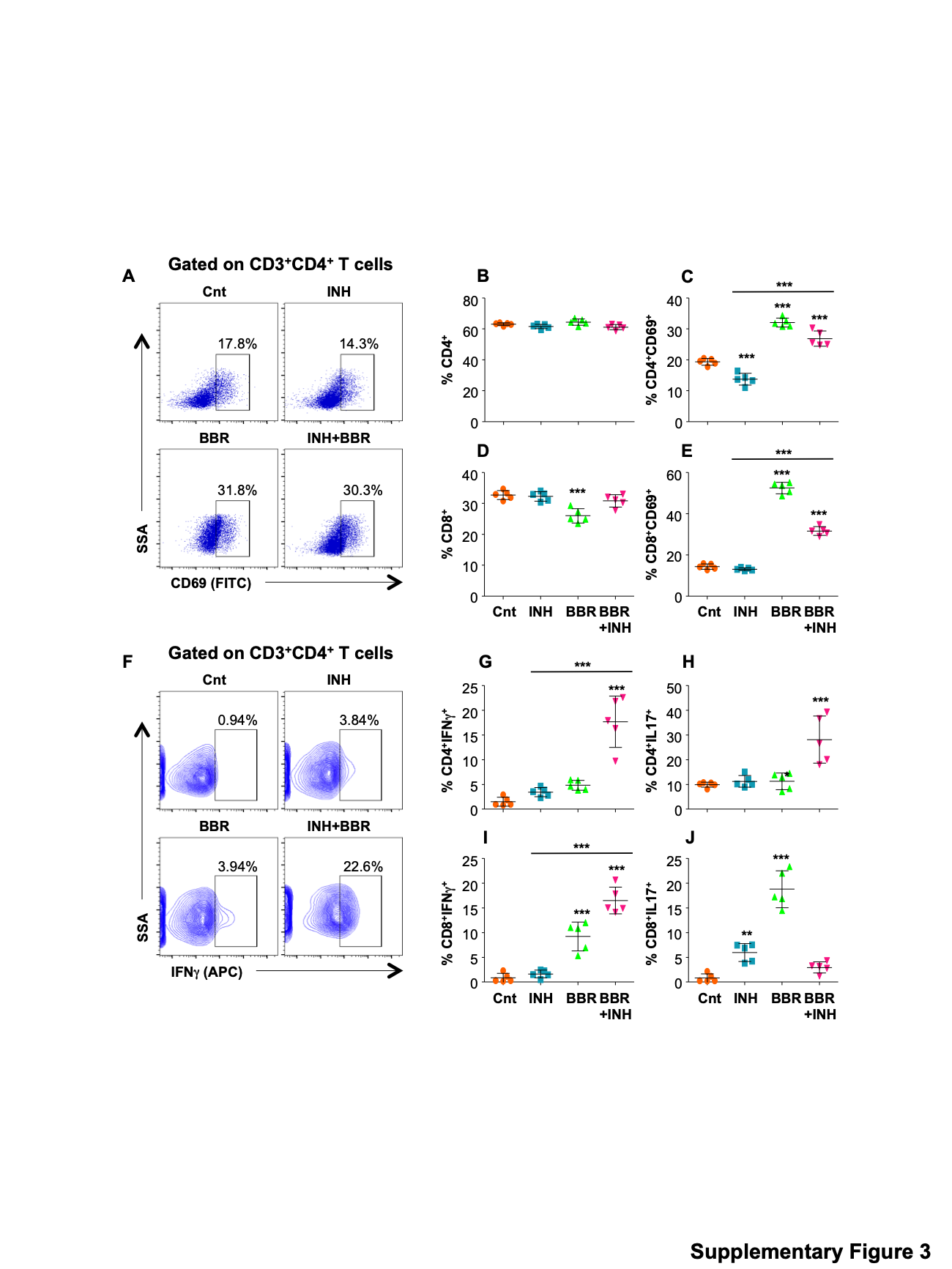

Supplement: S3 Fig — (A-E) Ex vivo stimulated splenocytes were surface stained with α-CD3 (Pacific Blue), α-CD4 (PerCPCy5.5), α-CD8 (APCCy7) and α-CD69 (FITC) followed by flow cytometry. (A) FACS dot plots and the percentage of (B) CD4+, (C) CD4+CD69+, (D) CD8+ and (E) CD8+CD69+ T cells in the spleen of infected mice. (F-J) Ex vivo stimulated splenocytes treated with monensin and brefeldin A for 2h and surface stained with α-CD3 (Pacific Blue), α-CD4 (PerCPCy5.5) and α-CD8 (APCCy7) followed by intracellular staining with α-IFNγ (APC) and α-IL17 (PECy7). (F) Representative dot plots and the percentage of (G) CD4+IFNγ+, (H) CD4+IL17+, (I) CD8+INFγ+ and (J) CD8+IL17+ cells in the infected spleen. Data is representative of two independent experiments. The data values represent mean ± SD (n = 5). *p<0.05, **p<0.005, ***p<0.0005. (TIFF) [file ppat.1011165.s003.tiff]

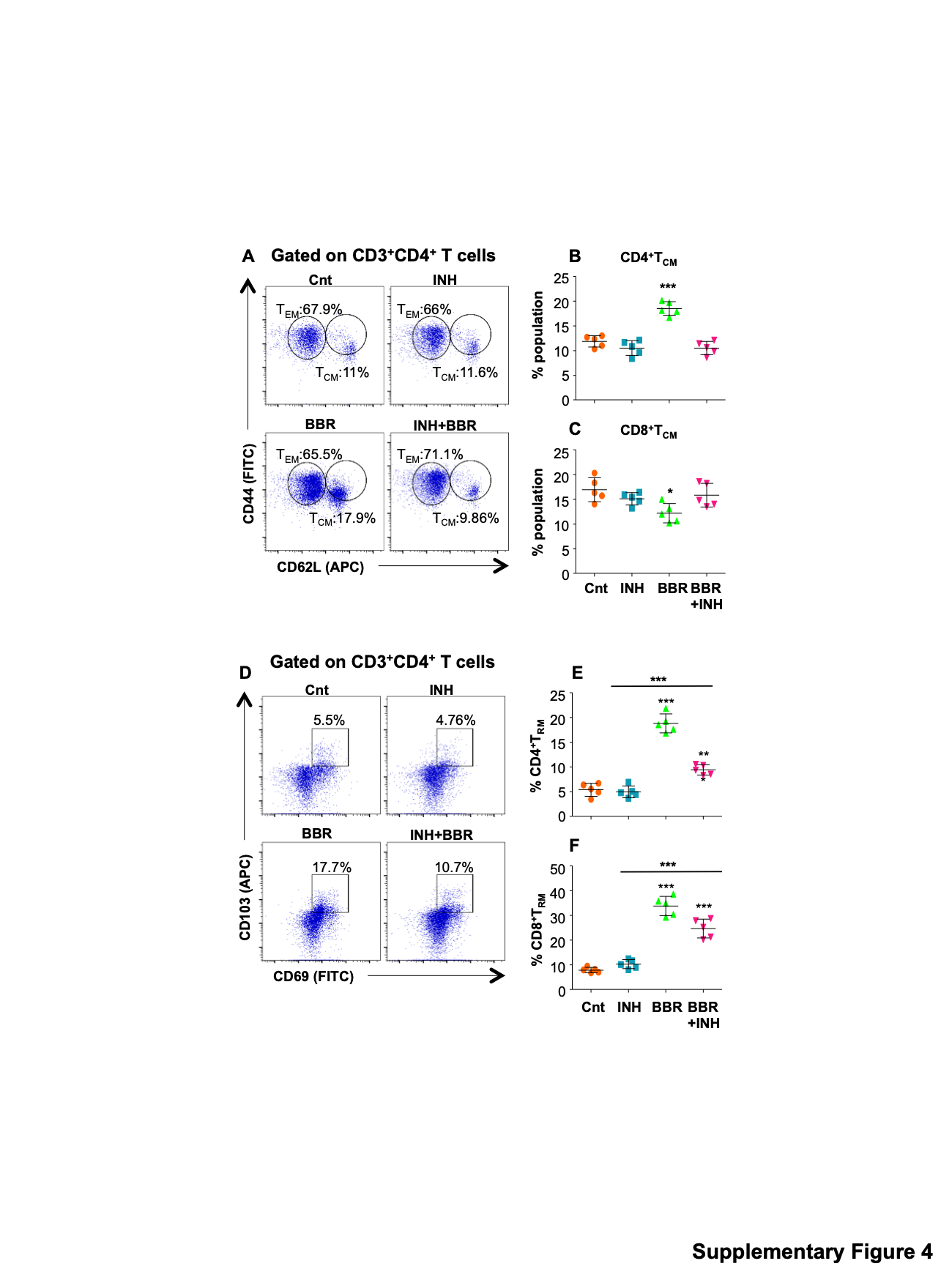

Supplement: S4 Fig — Ex vivo stimulated splenocytes were surface stained with α-CD3 (Pacific Blue), α-CD4 (PerCPCy5.5), α-CD8 (APCCy7), α-CD62L (APC) and α-CD44 (FITC) followed by flow cytometry. (A) Representative dot plots and the percentage of (B) CD4+ TCM cells and (C) CD8+ TCM cells in the infected spleen. (D-F) TRM cells were analysed by staining the splenocytes with α-CD3 (Pacific Blue), α-CD4 (PerCPCy5.5), α-CD8 (APCCy7), α-CD69 (FITC) and α-CD103 (APC) followed by flow cytometry. (D) Representative scatter dot-plot images and the percentage of (E) CD4+ TRM cells and (F) CD8+ TRM cells in the spleen of infected mice. Data is representative of two independent experiments. The data values represent mean ± SD (n = 5). *p<0.05, **p<0.005, ***p<0.0005. (TIFF) [file ppat.1011165.s004.tiff]

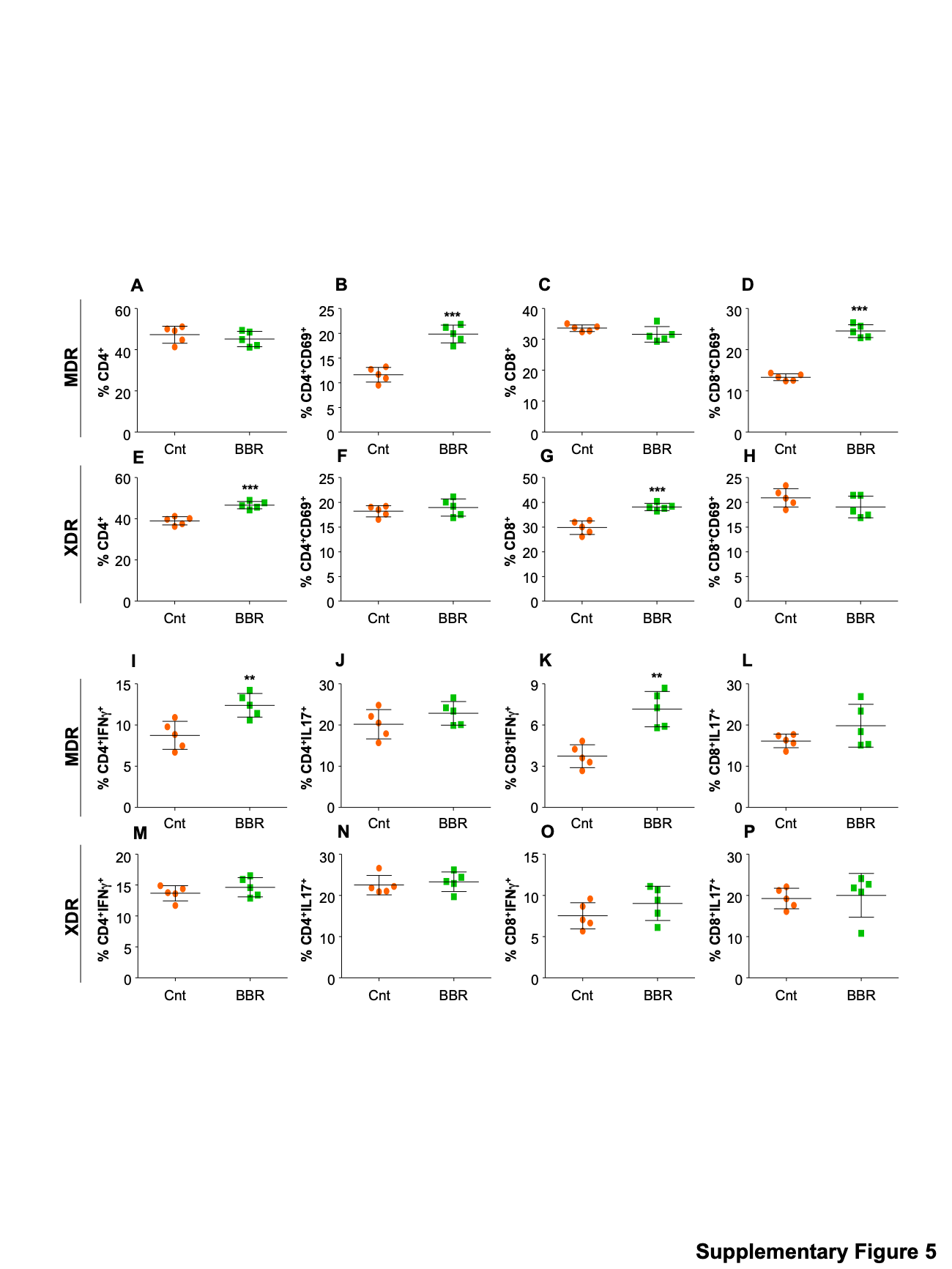

Supplement: S5 Fig — (A-H) Ex vivo stimulated lung cells isolated from the mice infected with MDR and XDR M.tb were surface stained with antibodies against CD3 (Pacific Blue), CD4 (PerCPCy5.5), CD8 (APCCy7) and CD69 (FITC) followed by flow cytometry. Percentage of CD4+, CD4+CD69+, CD8+, CD8+CD69+ T cells in the lungs of mice infected with (A-D) MDR TB and (E-H) XDR TB. (I-P) Stimulated lung cells were treated with monensin and brefeldin A followed by surface staining with α-CD3 (Pacific Blue), α-CD4 (PerCPCy5.5) and α-CD8 (APCCy7) and intracellular staining with α-IFNγ (APC) and α-IL17 (PECy7). Percentage of CD4+INFγ+, CD4+IL17+, CD8+IFNγ+, CD8+IL17+ T cells in the lungs of mice infected with (I-L) MDR and (M-P) XDR strains of M.tb. Data is representative of two independent experiments. The data values represent mean ± SD (n = 5). *p<0.05, **p<0.005, ***p<0.0005. (TIFF) [file ppat.1011165.s005.tiff]

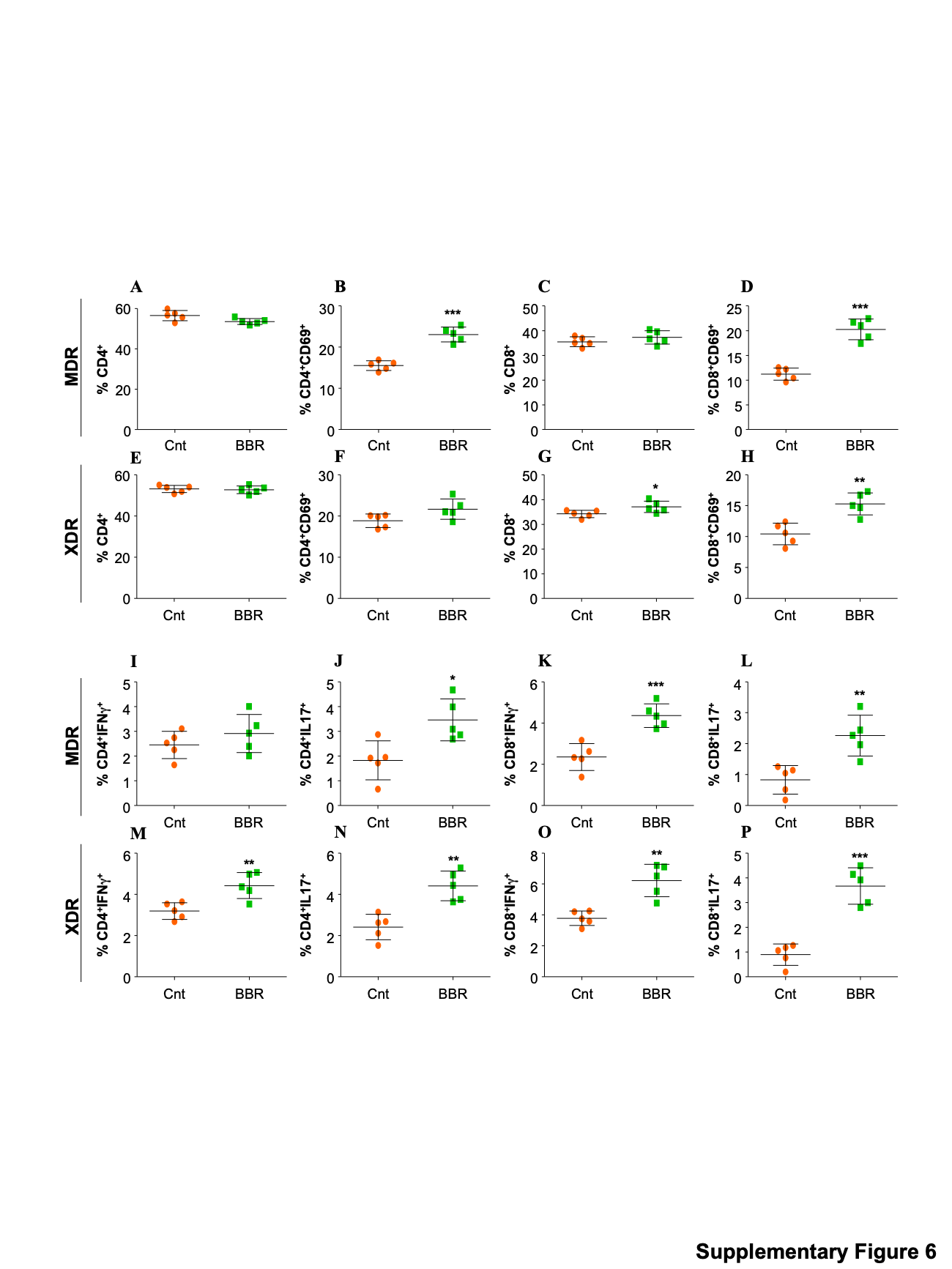

Supplement: S6 Fig — Ex vivo stimulated splenocytes isolated from MDR and XDR infected mice were analysed for T cell responses as described earlier. Percentage of CD4+, CD4+CD69+, CD8+ and CD8+CD69+ T cells in the spleen of mice infected with (A-D) MDR and (E-H) XDR strains of M.tb. Percentage of CD4+INFγ+, CD4+IL17+, CD8+IFNγ+ and CD8+IL17+ T cells in the spleen of mice infected with (I-L) MDR and (M-P) XDR strains of M.tb. Data is representative of two independent experiments. The data values represent mean ± SD (n = 5). *p<0.05, **p<0.005, ***p<0.0005. (TIFF) [file ppat.1011165.s006.tiff]

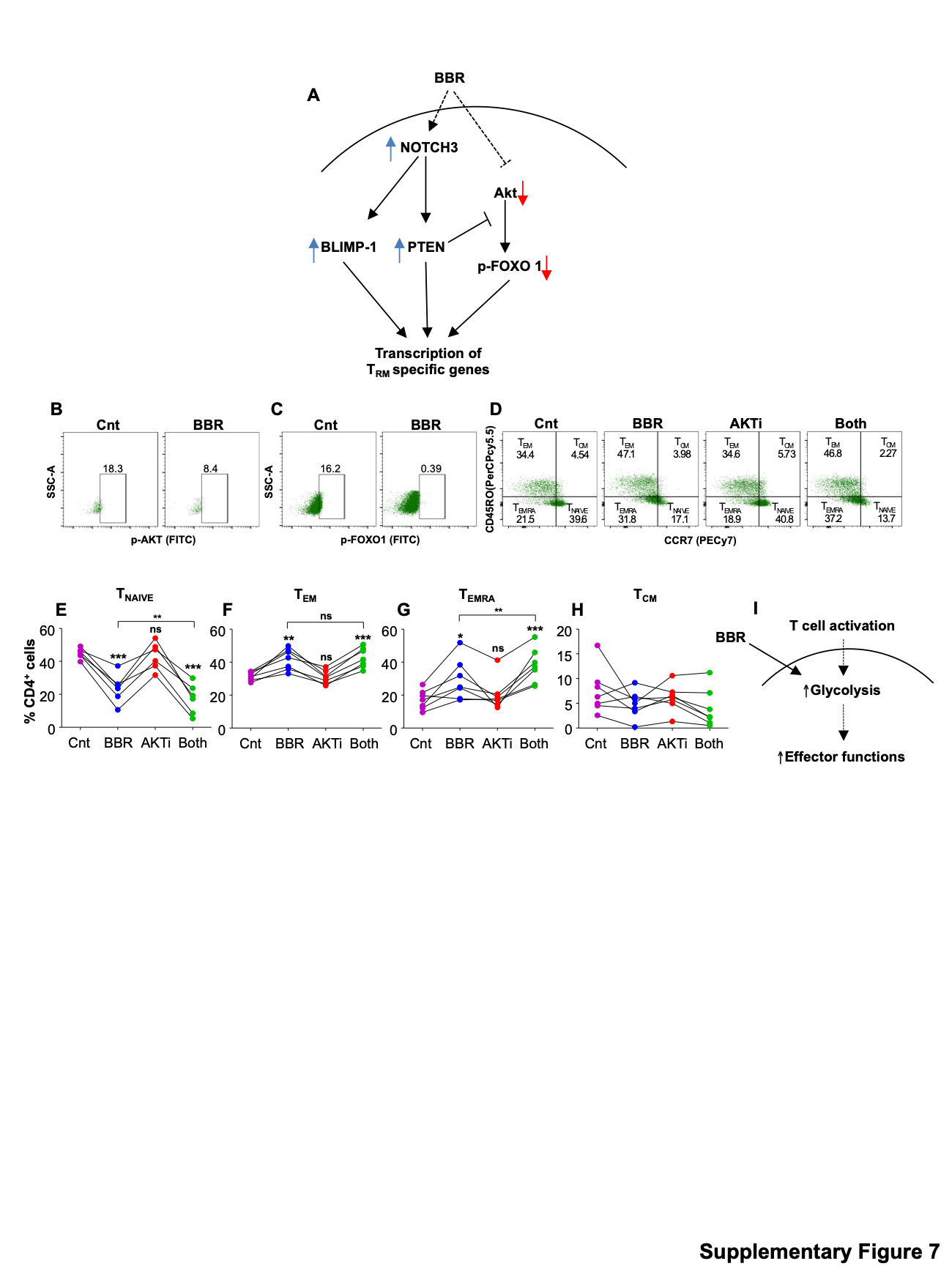

Supplement: S7 Fig — (A) Flowchart depicting the critical components involved in the transcription of T cell resident memory-specific genes. FACS plots from a single donor representing the percentage of CD4+ T cells expressing (B) p-AKT and (C) p-FOXO1. (D) Representative FACS scatter plots and the percentage of (E) CD4+ TNAIVE cells, (F) CD4+ TEM cells, (G) CD4+ TEMRA cells and (H) CD4+ TCM cells in the human PBMCs treated with BBR and AKTi. (I) Proposed model of enhanced T cell effector functions upon BBR treatment. The data values represent mean ± SD (n is 7). *p<0.05, **p<0.005, ***p<0.0005. (TIFF) [file ppat.1011165.s007.tiff]

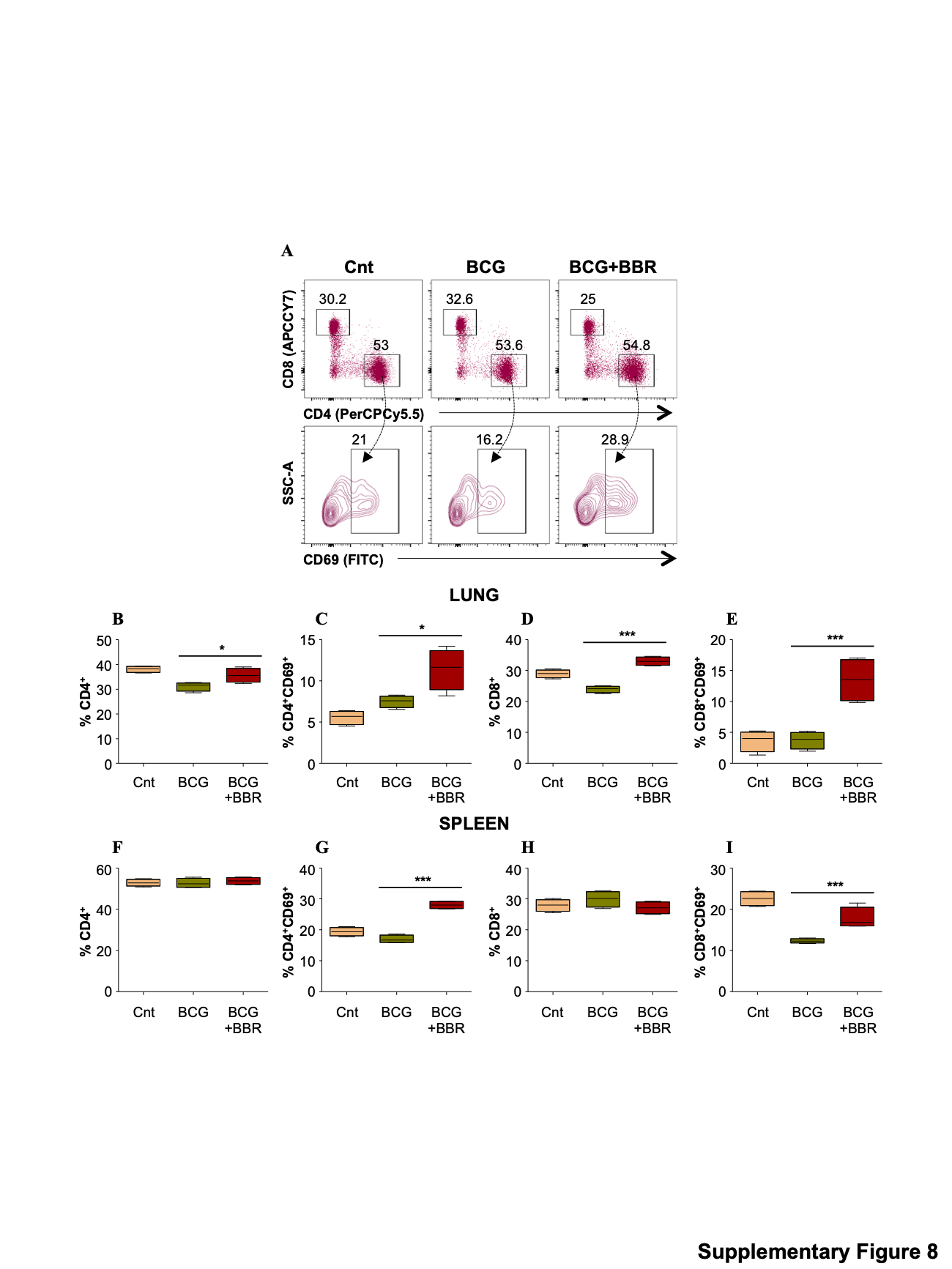

Supplement: S8 Fig — The lungs and the spleen of control, BCG and BCG-BBR vaccinated animals were harvested and analysed for T cell activation as described before. (A) Representative FACS plots and scatter plots depicting the percentage of CD4+, CD4+CD69+, CD8+ and CD8+CD69+ T cells in (B-E) the lungs and (F-I) the spleen of vaccinated animals before M.tb challenge. Data is representative of two independent experiments. The data values represent mean ± SD (n = 4). *p<0.05, **p<0.005, ***p<0.0005. (TIFF) [file ppat.1011165.s008.tiff]

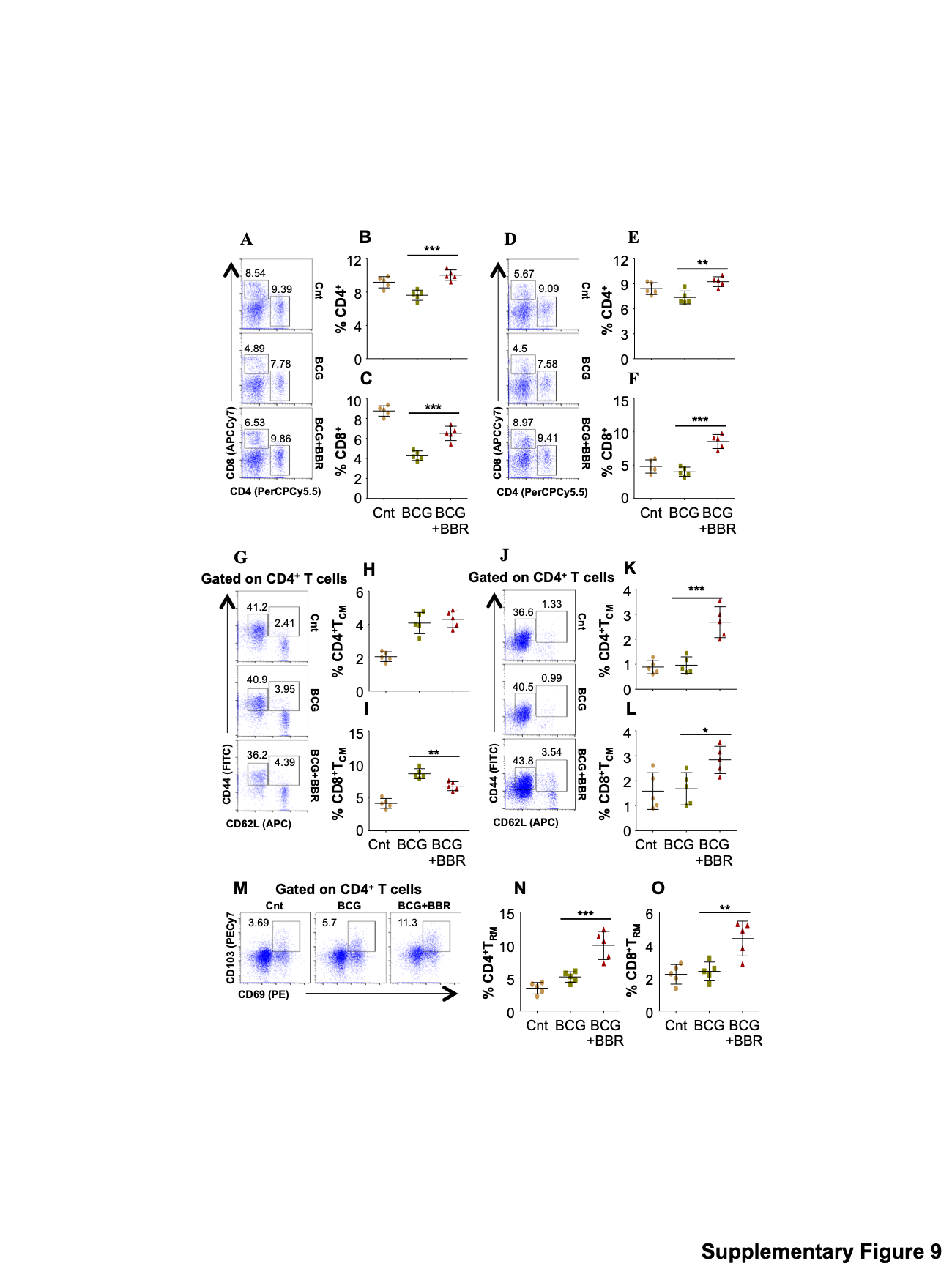

Supplement: S9 Fig — Percentage of CD4+ and CD8+ T cells in (A-C) the lungs and (D-F) the spleen of infected animals. Percentage of CD4+ TCM cells and CD8+ TCM cells in (G-I) the lungs and (J-L) the spleen of infected animals. (M) FACS plots and quantification of (N) CD4+ TRM cells and (O) CD8+ TRM cells in the spleen of infected animals. Data is representative of two independent experiments. The data values represent mean ± SD (n = 5). *p<0.05, **p<0.005, ***p<0.0005. (TIFF) [file ppat.1011165.s009.tiff]

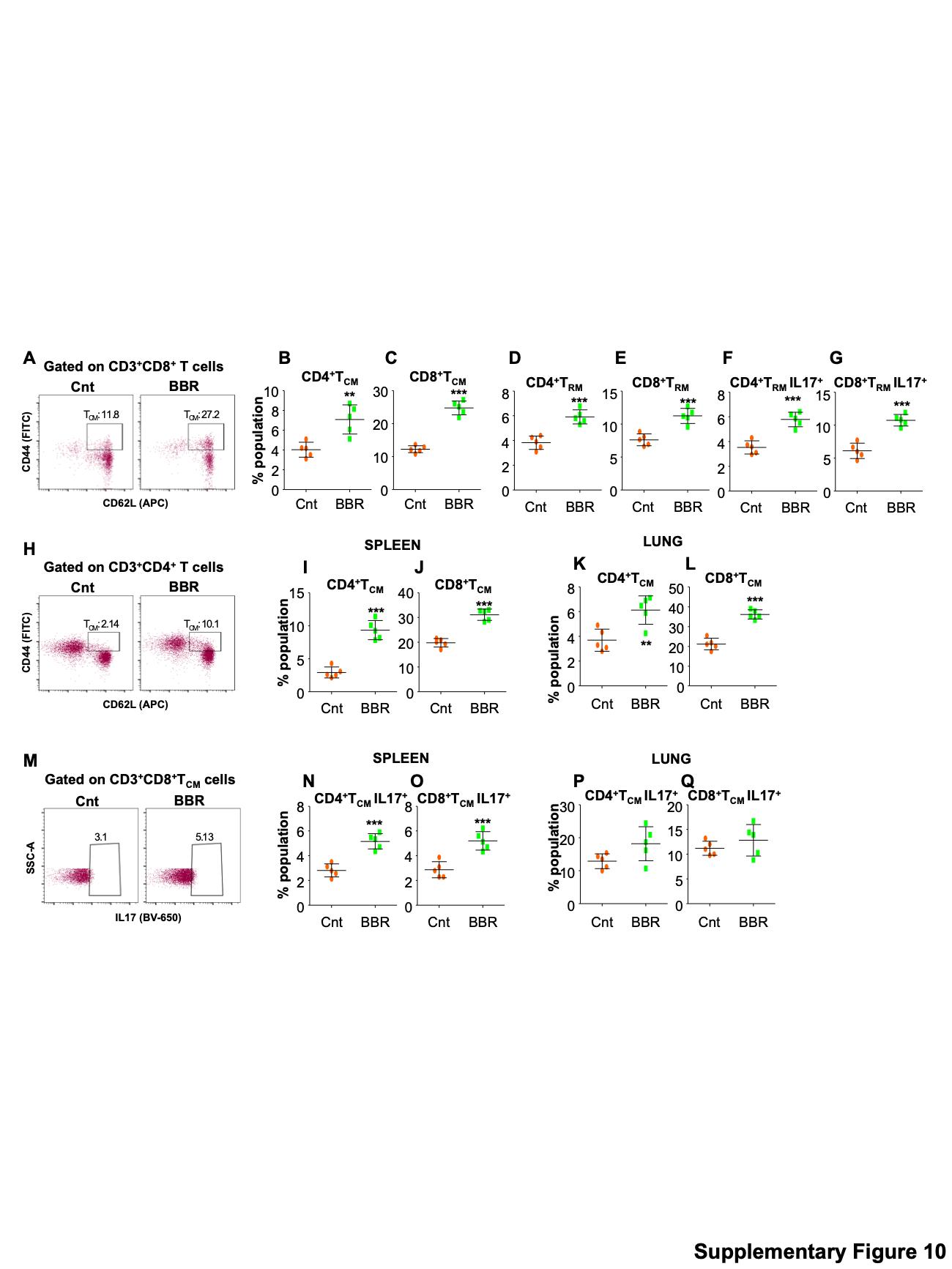

Supplement: S10 Fig — (A) FACS plots representing the percentage of (B) CD4+ TCM cells and (C) CD8+ TCM cells in the lungs of re-activation group mice. Percentage of (D) CD4+ TRM cells, (E) CD8+ TRM cells, (F) CD4+IL17+ TRM cells and (G) CD8+IL17+ TRM cells in the spleen of re-infected mice. (H) FACS plots representation and the percentage of CD4+ TCM cells and CD8+ TCM cells in (I&J) the spleen and (K&L) the lungs of re-infected mice. (M-Q) Percentage of central memory T cells producing IL17. (M) Representative FACS scatter plots of and the percentage ofCD4+IL17+ TCM cells andCD8+IL17+ TCM cells in (N&O) the spleen and (P&Q) the lungs of re-infected mice. Data is representative of two independent experiments. The data values represent mean ± SD (n is 5). *p<0.05, **p<0.005, ***p<0.0005. (TIFF) [file ppat.1011165.s010.tiff]
