# Supplementary material for: Berberine governs NOTCH3/AKT signaling to enrich lung-resident memory T cells during tuberculosis
Source: PLoS Pathog. 2023 Mar 7;19(3):e1011165. doi: 10.1371/journal.ppat.1011165 (PMC9990925; doi:10.1371/journal.ppat.1011165)
Supplement: S1 Table — (DOCX) [file ppat.1011165.s011.docx]

**S1 Table: List of the Primers used in the study:**

| Primer | Sequence (5’-3’) |
| --- | --- |
| IFNγ Forward Primer | GCGTCATTGAATCACACCTG |
| IFNγ Reverse Primer | TGAGCTCATTGAATGCTTGG |
| CCR7 Forward Primer | TGTACGAGTCGGTGTGCTTC |
| CCR7 Reverse Primer | GGTAGGTATCCGTCATGGTCTTG |
| IL2 Forward Primer | AAGCTCTACAGCGGAAGCAC |
| IL2 Reverse Primer | ATCCTGGGGAGTTTCAGGTT |
| CXCR4 Forward Primer | GACTGGCATAGTCGGCAATG |
| CXCR4 Reverse Primer | AGAAGGGGAGTGTGATGACAAA |
| TNFα Forward Primer | TAGCCAGGAGGGAGAACAGA |
| TNFα Reverse Primer | TTTTCTGGAGGGAGATGTGG |
| IL10 Forward Primer | CATGGGTCTTGGGAAGAGAA |
| IL10 Reverse Primer | AACTGGCCACAGTTTTCAGG |
| IL6 Forward Primer | CCGGAGAGGAGACTTCACAG |
| IL6 Reverse Primer | TCCACGATTTCCCAGAGAAC |
| IL4 Forward Primer | CCAAGGTGCTTCGCATATTT |
| IL4 Reverse Primer | ATCGAAAAGCCCGAAAGAGT |
| IL1β Forward Primer | CCCAAGCAATACCCAAAGAA |
| IL1β Reverse Primer | GCTTGTGCTCTGCTTGTGAG |
| IL22 Forward Primer | CCGAGGAGTCAGTGCTAAGG |
| IL22 Reverse Primer | CATGTAGGGCTGGAACCTGT |
| IL23 Forward Primer | AATAATGTGCCCCGTATCCA |
| IL23 Reverse Primer | AGGCTCCCCTTTGAAGATGT |
| IL17A Forward Primer | TTCAGGGTCGAGAAGATGCT |
| IL17A Reverse Primer | AAACGTGGGGGTTTCTTAGG |
| IL12p40 Forward Primer | AAGGAACAGTGGGTGTCCAG |
| IL12p40 Reverse Primer | GGAGACACCAGCAAAACGAT |
| CCL2 Forward Primer | AGCACCAGCCAACTCTCACT |
| CCL2 Reverse Primer | CGTTAACTGCATCTGGCTGA |
| GAPDH Forward Primer | AACTTTGGCATTGTGGAAGG |
| GAPDH Reverse Primer | GGATGCAGGGATGATGTTCT |
| APH1A Human Forward Primer | TTTTTCGGCTGCACTTTCGTC |
| APH1A Human Reverse Primer | TGCGACCAGGATGATAACGC |
| FOXO1 Human Forward Primer | GGCTGCCGCGATCATAGAC |
| FOXO1 Human Reverse Primer | GGCTGGTTAGCGATCTCTGG |
| NOTCH3 Human Forward Primer | TGGCGACCTCACTTACGACT |
| NOTCH3 Human Reverse Primer | CACTGGCAGTTATAGGTGTTGAC |
| PRDM1 Human Forward Primer | AAGCAACTGGATGCGCTATGT |
| PRDM1 Human Reverse Primer | GGGATGGGCTTAATGGTGTAGAA |
| PSEN1 Human Forward Primer | GACGACCCCAGGGTAACTC |
| PSEN1 Human Reverse Primer | ACTGACTTAATGGTAGCCACGA |
| PTEN Human Forward Primer | TTTGAAGACCATAACCCACCAC |
| PTEN Human Reverse Primer | ATTACACCAGTTCGTCCCTTTC |
| 18S_rRNA Human Forward Primer | GCTTAATTTGACTCAACACGGGA |
| 18S_rRNA Human Reverse Primer | AGCTATCAATCTGTCAATCCTGTC |
